# Joint synthesis of multiple correlated outcomes in networks of interventions

**DOI:** 10.1093/biostatistics/kxu030

**Published:** 2014-07-02

**Authors:** Orestis Efthimiou, Dimitris Mavridis, Richard D. Riley, Andrea Cipriani, Georgia Salanti

**Affiliations:** Department of Hygiene and Epidemiology, University of Ioannina School of Medicine, 1186 Ioannina 45110, Greece; Department of Hygiene and Epidemiology, University of Ioannina School of Medicine, 1186 Ioannina 45110, Greece; Department of Primary Education, University of Ioannina, 1186 Ioannina 45110, Greece; School of Health and Population Sciences, University of Birmingham, Edgbaston, Birmingham, B152TT, UK; Department of Psychiatry, University of Oxford, Warneford Hospital, Oxford, OX37JX, UK WHO Collaborating Centre for Research and Training in Mental Health and Service Evaluation, Department of Public Health and Community Medicine, Section of Psychiatry, University of Verona, Policlinico Giambattista Rossi, Piazzale L.A. Scuro 10, 37134 Verona, Italy; Department of Hygiene and Epidemiology, University of Ioannina School of Medicine, 1186, Ioannina, 45110, Greece

**Keywords:** Correlation, Heterogeneity, Mixed-treatment comparison, Multivariate meta-analysis

## Abstract

Multiple outcomes multivariate meta-analysis (MOMA) is gaining in popularity as a tool for jointly synthesizing evidence coming from studies that report effect estimates for multiple correlated outcomes. Models for MOMA are available for the case of the pairwise meta-analysis of two treatments for multiple outcomes. Network meta-analysis (NMA) can be used for handling studies that compare more than two treatments; however, there is currently little guidance on how to perform an MOMA for the case of a network of interventions with multiple outcomes. The aim of this paper is to address this issue by proposing two models for synthesizing evidence from multi-arm studies reporting on multiple correlated outcomes for networks of competing treatments. Our models can handle continuous, binary, time-to-event or mixed outcomes, with or without availability of within-study correlations. They are set in a Bayesian framework to allow flexibility in fitting and assigning prior distributions to the parameters of interest while fully accounting for parameter uncertainty. As an illustrative example, we use a network of interventions for acute mania, which contains multi-arm studies reporting on two correlated binary outcomes: response rate and dropout rate. Both multiple-outcomes NMA models produce narrower confidence intervals compared with independent, univariate network meta-analyses for each outcome and have an impact on the relative ranking of the treatments.

## 1. Introduction

When studies report on multiple outcomes for each patient, a joint, multivariate meta-analysis (multiple outcomes multivariate meta-analysis, MOMA) can be used to incorporate all possible correlations in order to perform a simultaneous synthesis of all data for all outcomes. The effects of disregarding all possible correlations by performing a series of independent, univariate analyses have been explored and may include a loss of precision and an increase of selective reporting bias (Kirkham *and others*, 2012; Riley, 2009). There are two types of correlations to consider: within-study correlations of the estimated relative treatment effects on the multiple outcomes, reflecting the fact that the same patients report on each of these outcomes and between-study correlations of the true relative treatment effects on the different outcomes, reflecting the way these true effects depend on each other when measured in different setting. Many estimation methods for MOMA models have been suggested in recent years, both in a frequentist and a Bayesian setting (Jackson *and others*, 2013; Riley *and others*, 2007a; Wei and Higgins, 2013b; for reviews, see Jackson *and others*, 2011; Mavridis and Salanti, 2013).

Currently available models for performing an MOMA of randomized trials focus on the case of meta-analysis for studies that compare only two treatments and report on multiple, possibly correlated outcomes. As in practice, many alternative treatments exist for the same condition, network meta-analysis (NMA) is increasingly gaining in popularity as it allows the synthesis of data over a network of treatments compared in studies for a certain outcome (Dias *and others*, 2013; Lu and Ades, 2004; Salanti, 2012; Salanti *and others*, 2008). It is therefore desirable to extend MOMA methods for multiple-treatment comparisons. To our knowledge, there is no general model available for performing a joint, multiple-outcome NMA for multi-arm studies, that incorporates both within- and between-studies correlations (multiple outcomes network meta-analysis, MONMA), for binary, continuous and time-to-event outcomes. In this paper, we propose two different modeling approaches to perform such an analysis. The first approach is based on making a set of simplifying assumptions to model both within- and between-studies correlation coefficients. The second approach is a generalization of a bivariate model proposed by Riley *and others* that allows for a single correlation coefficient to model the overall correlation, an amalgam of the within- and between-study correlations (Riley *and others*, 2008). We fit the models in a Bayesian framework that offers flexibility in incorporating prior beliefs and allows for a straightforward inclusion of studies that do not report on all outcomes, as well as accounting for uncertainty in parameter estimates.

The paper is structured as follows: in Section 2, we describe the data we used to illustrate our methods. In Section 3, we present the two approaches and discuss the technicalities of fitting the models. In Section 4, we apply the methods to our data to produce estimates for outcome-specific relative treatment effects and evaluate the relative ranking of the treatments for each outcome. In Section 5, we present our conclusions, discuss the limitations of the models and suggest areas for future research.

## 2. Example: the acute mania dataset

The dataset we used as an example is a network of 68 studies comparing 13 active antimanic drugs and placebo for acute mania (Cipriani *and others*, 2011). The majority of the studies had two arms (50 studies) and there were 18 three-arm studies. The primary outcomes of interest were efficacy and treatment discontinuation (acceptability) after 3 weeks. Acceptability was estimated as the number of patients leaving the study early for any reason, before or after having a response to the treatment, out of the total number of randomized patients. All-cause discontinuation from allocated treatment may be due to a number of reasons, such as adverse effects, inefficacy, other reasons not related to treatment (e.g. moving away, protocol violation), or a combination of the above. Efficacy was reported either as dichotomous outcome (number of patients who responded to treatment, defining response as a reduction of at least 50% in manic symptoms from baseline to week 3) or as continuous outcome (mean change scores on a standardized rating scale for mania after 3 weeks). Although we recognize that outcome dichotomization may lose some information, in this paper we use data on efficacy as a dichotomous outcome as it may be easier to interpret clinically and allows us to illustrate our methodology for two related binary outcomes, a frequent scenario encountered by researchers. Only a few patients did not provide data for response to treatment and their outcome was coded as treatment failure; an imputation assumption that has been shown to be sensible (Spineli *and others*, 2013). Among the included studies, only 65 contributed with data for at least one of the outcomes of interest: 18 studies (28%) did not report usable data on response, while only one study did not report information on the number of dropouts (1.5%). Efficacy and acceptability outcomes are generally expected to be negatively correlated; although early full response to the treatment may be a cause for leaving the study prematurely, more often it is reasonable to assume that more efficacious treatments are associated with a lower dropout rate. Within-study correlations were not reported in any of the studies and individual patient data (IPD) which could be used to estimate within-study correlations were not available. The dataset included a total of 69 head-to-head comparisons for response and 100 for dropout. In Section 1 of the supplementary material available at *Biostatistics* online, we provide a table with all head-to-head comparisons for each outcome, along with the odds ratios and their 95% confidence interval. The initial analysis consisted of two independent network meta-analyses, one for each outcome (Cipriani *and others*, 2011). As both outcomes are crucially important for clinical decision making, the ranking of the treatments was presented for both efficacy and acceptability in a two-dimensional scatter plot (Figure 6 in Cipriani *and others*) so that efficacious treatments with high tolerability could be identified. This is a suboptimal approach and the rankings of the treatments for each outcome can be better estimated jointly in a MONMA model to account for the correlation in the outcomes. This is especially important here as 19 studies provide data on only one of the two outcomes, and MONMA can “borrow strength” from these studies even for the missing outcomes.

## 3. Methods

First, we revise the models used for performing an MOMA when only two treatments are compared. We then generalize these methods for a network of interventions that includes multi-arm studies.

### 3.1 Pairwise multivariate meta-analysis of multiple outcomes

Suppose we have a total of $N_{{S}}$ studies comparing two treatments (e.g. a new treatment versus a placebo) with respect to two different but correlated outcomes, denoted with $R$ and $D$. We denote the observed treatment effects in study for outcomes $R$ and $D$ with $y_{i,R}$ and $y_{i,D}$ respectively; in our example, these are the log odds ratio estimates comparing the two treatments for $R$ and $D$, but in other situations they could be mean difference or log hazard ratio estimates, for example. The bivariate random effects meta-analysis model is:

(3.1) \begin{equation*}\label{eq3.1} \left(\begin{matrix} y_{1,R} \\ y_{1,D} \\ y_{2,R} \\ \vdots \\ y_{N_S ,D} \end{matrix}\right)=\left(\begin{matrix} 1 & 0 \\ 0 & 1 \\ 1 & 0 \\ \vdots & \vdots \\ \end{matrix}\right)\times \left(\begin{matrix} \beta _R \\ \beta _D \\ \end{matrix}\right)+\left(\begin{matrix} \delta _{1,R} \\ \delta _{1,D} \\ \delta _{2,R} \\ \vdots \\ \delta _{N_S ,D} \\ \end{matrix}\right)+\left(\begin{matrix} \varepsilon _{1,R} \\ \varepsilon _{1,D} \\ \varepsilon _{2,R} \\ \vdots \\ \varepsilon _{N_S ,D} \\ \end{matrix}\right). \end{equation*}

The parameters $\beta _R$ and $\beta _D$ are the true mean relative treatment effects, which summarize how the treatment performs on average across studies for each outcome. We can rewrite (3.1) using matrix notation as $\boldsymbol{Y} = \boldsymbol{X}\boldsymbol {\beta }+\boldsymbol {\delta }+\boldsymbol {\varepsilon }$. Assuming all studies report both outcomes, $\boldsymbol{Y}$ is the 2$N_{{S}}$-imensional vector of the observed effects, $\boldsymbol {\beta }$ is the vector $(\beta _R,\beta _D)$, ***X*** is the ($N_{S}\times 2$) design matrix, and $\boldsymbol {\delta }$ and $\boldsymbol {\varepsilon }$ are the vectors of random effects (reflecting between-study variability) and random errors (reflecting within-study sampling variability), respectively. In the meta-analysis model of (3.1), we must incorporate the correlations between the outcomes, both within and between studies. We assume multivariate normal distributions for $\boldsymbol {\varepsilon }$ and $\boldsymbol {\delta }$, so that and $\boldsymbol {\varepsilon } \sim N (0, \boldsymbol {\Sigma })$ and $\delta \sim N(0,\boldsymbol {\varDelta })$, with $\boldsymbol {\Sigma }$ and $\boldsymbol {\varDelta }$ being the within- and between-study variance–covariance matrices. The variance–covariance matrix for the random effects takes a block-diagonal form:

(3.2) \begin{equation*}\label{eq3.2} \boldsymbol{\varDelta} =\left(\begin{matrix} \tau _R ^2 & \rho ^\tau \tau _R \tau _D & 0 & 0& \cdots \\ \rho ^\tau \tau _R \tau _D & \tau _D ^2 & 0 & 0& \ldots \\ 0 & 0 & \tau _R ^2 & \rho ^\tau \tau _R \tau _D & \ldots \\ 0 & 0 & \rho ^\tau \tau _R \tau _D & \tau _D ^2 & \ldots \\ \vdots & \vdots & \vdots & \vdots & \ddots\\ \end{matrix}\right)=\left(\begin{matrix} \boldsymbol{\varDelta}_{(2\times 2)} & 0 & \cdots \\ 0 &\boldsymbol{\varDelta}_{(2\times 2)} & \cdots \\ \vdots & \vdots & \ddots \\ \end{matrix}\right). \end{equation*}

The above $2N_{{S}} \times 2N_{S}$ matrix involves the heterogeneity standard deviations for each outcome, $\tau _R$ and $\tau _D$, and the between-studies correlation coefficient, $\rho ^\tau $. Note that this between-studies variance–covariance matrix is block-diagonal with identical $\boldsymbol {\Delta }_{(2\times 2)}$ matrices in its diagonal. The parameters $\rho ^\tau $, $\tau _R$, and $\tau _D$ need to be estimated from the model. In a frequentist framework options include restricted maximum likelihood and methods of moments; here, we focus on a Bayesian framework estimated using Markov Chain Monte Carlo (described in Section 4). The random errors variance–covariance matrix is also block diagonal:

(3.3) \begin{equation*}\label{eq3.3} \boldsymbol{\varSigma} =\left(\begin{matrix} \sigma _{1,R}^2 & \rho _1 \sigma _{1,R} \sigma _{1,D} &0 & 0 & \cdots \\ \rho _1 \sigma _{1,R} \sigma _{1,D} & \sigma _{1,D}^2 &0 & 0 & \ldots \\ 0 & 0 & \sigma _{2,R}^2 & \rho _2 \sigma _{2,R}\sigma _{2,D} & \ldots \\ 0 & 0 & \rho _2 \sigma _{2,R} \sigma _{2,D} &\sigma _{2,D}^2 & \ldots \\ \vdots & \vdots & \vdots & \vdots & \ddots\\ \end{matrix}\right)=\left(\begin{matrix} \varSigma_1 & 0 & \cdots \\ 0 &\varSigma_2 & \cdots \\ \vdots & \vdots & \ddots \\ \end{matrix}\right). \end{equation*}

In this matrix, $\rho _i$ is the within-study correlation coefficient, and $\sigma _{i,R}^2, \sigma _{i,D}^2$ are the variances of the effect sizes in each study $i$. All entries in $\boldsymbol {\varSigma }$ are estimated from the data. Sample estimates for $\sigma _{i,R}^2$ and $\sigma _{i,D}^2$ are often available, but few studies, if any, would provide enough information to estimate the within-study correlation coefficient $\rho _{i}$ and the majority of meta-analyses do not have access to IPD that would enable its estimation. Within a Bayesian framework, we can give prior distributions to all the correlation coefficients entering (3.3) in order to perform a full multivariate meta-analysis. One can model these coefficients in a variety of ways, e.g. assume all $\rho _{i}$ to be equal $(\rho _{i} = \rho \forall i)$, assume a different coefficient depending on study characteristics, place a vague or informative prior on each $\rho _{i}$, etc.

Following a different approach, Riley *and others* (2008) proposed an alternative model for bivariate random effects pairwise meta-analysis that allows for a single coefficient to model the overall correlation, an amalgam of the correlations within and between studies. Instead of modeling $\boldsymbol {\varSigma }$ and $\boldsymbol {\varDelta }$ separately, they assume an overall variance–covariance matrix $\boldsymbol {\varOmega }$, so that $\boldsymbol{Y}=\boldsymbol{X}\boldsymbol {\beta } +\boldsymbol {\eta }$ with $\boldsymbol {\eta }\sim N(0,\boldsymbol {\varOmega })$. This matrix $\boldsymbol {\varOmega }$ is again block diagonal with each block corresponding to a study, so that $\boldsymbol {\varOmega }=Diag(\boldsymbol {\varOmega }_{\textbf {1}}$, $\boldsymbol {\Omega }_{\textbf {2}},\ldots ,\boldsymbol {\Omega }_{\boldsymbol{N}_{\boldsymbol{S}}})$ For a study $i$,

(3.4) \begin{equation*}\label{eq3.4} \boldsymbol{\varOmega}_{i} =\left(\begin{matrix} \psi_{R}^{2}+\sigma_{i,R}^{2} &\rho_{i}^{h} \sqrt{\left(\psi_{R}^{2}+\sigma_{i,R}^{2}\right)\left(\psi_{D}^{2}+\sigma _{i,D}^{2}\right)} \\ \rho_{i}^{h} \sqrt{\left(\psi_{R}^{2}+\sigma_{i,R}^{2} \right)\left(\psi_{D}^{2}+\sigma_{i,D}^{2}\right)}& \psi _{D}^{2}+\sigma_{i,D}^{2} \\ \end{matrix}\right). \end{equation*}

The $\rho _{i}^{h}$ coefficient in (3.4) is the overall correlation in study $i$, a hybrid of the within- and between-study correlation coefficients. We can again model the different $\rho _{i}^{h}$ in a variety of ways, depending on the nature of the data, e.g. $\rho _{i}^{h}=\rho \,\forall \,i$. The $\psi $ parameters model for the variation additional to the sampling error that enters due to heterogeneity, and they are similar to the $\tau $ parameters of (3.2), but not directly equivalent unless the within-study variances are small relative to the between-study variances in model (3.2). The clear advantage of model (3.4) is that the within-study correlations are no longer needed.

### 3.2 NMA for two correlated outcomes

The two models described in the previous section can be easily extended to perform a meta-analysis for a network of treatments, if all included studies have just two treatments arms. These models, however, cannot handle the case of studies comparing more than two treatments.

In this section, we present two models for performing an NMA of studies with multiple arms reporting on two correlated outcomes, generalizing the models presented in Section 3.1. The outcomes can be binary (and relative treatment effect can be measured as log odds ratios or log risk ratios), continuous (effects measured as mean differences or standardized mean differences) or time to event (effects measured as log hazard ratios). Note that in order to use the standardized mean difference for a continuous outcome a large sample approximation is required. For more details, see Section 3 of supplementary material available at *Biostatistics* online. In the acute mania example, the outcomes are identified as the binary response to the treatment ($R$) and dropout rate ($D$). We exemplify the methodology for the case of networks containing studies with a maximum of three arms. We assume a random effects model and that the consistency equations ($\beta _{XY,R}=\beta _{XZ,R}-\beta _{YZ,R})$ hold for all treatments $X,Y$ and $Z$; similarly for outcome $D$.

#### 3.2.1 Model 1: Simplifying the variance–covariance matrices

The first method is based on simplifying the within- and between-study variance–covariance matrices so that the number of parameters needed is minimized, eases computational burden and potential estimation difficulties. Let us start by considering a network of studies reporting on the correlated outcomes $R$ and $D$ for a network of $N_{T}$ different treatments The model is $\boldsymbol{Y}=\boldsymbol{X}\boldsymbol {\beta } +\boldsymbol {\delta } +\boldsymbol {\varepsilon }$ with $\boldsymbol{Y}$ the vector of the observed effects, $\boldsymbol{X}$ the design matrix, $\boldsymbol {\beta }$ the vector of the basic parameters, i.e. the $N_{T}-1$ parameters for the comparison of each treatment versus the reference (Lu and Ades, 2004; Salanti *and others*, 2008), $\boldsymbol {\delta }$ the vector of random effects, and $\boldsymbol {\varepsilon }$ the vector of random errors (Dias *and others*, 2013; Salanti *and others*, 2008). The design matrix $\boldsymbol{X}$ describes the structure of the network and embeds the consistency equations (Salanti *and others*, 2008); it maps the observed comparisons into the basic parameters. For example, if $A$ is chosen to be the reference treatment, a study comparing $B$ to $C$ for outcome $R$ provides information for a linear combination of two basic parameters as $\beta _{BC,R}=\beta _{AC,R}-\beta _{AB,R}$.

For a two-arm study $i$ that compares treatments $A$ and $B$ the random errors are assumed to follow a multivariate normal distribution, $(\delta _{i,AB,R},\delta _{i,AB,D})'\sim N(0,\boldsymbol {\varDelta }_{(2\times 2)})$, with $\boldsymbol {\varDelta }_{(2\times 2)}$ defined in (3.2). Note that this matrix is always positive definite for $-1<\rho ^{\tau }<1$. In NMA, it is often assumed that the heterogeneity is independent of the comparison being made (Salanti *and others*, 2008); i.e $\tau _{AB,R}^{2}=\tau _{R}^{2}$ and $\tau _{AB,D}^{2}=\tau _{D}^{2}$ for every pair of treatments $A$, $B$, and we also assume this here. For a three-arm study $i$ that compares treatments $A$, $B$ and $C$, the random effects are again assumed to follow a multivariate normal distribution $(\delta _{i,AB,R},\delta _{i,AB,D}, \delta _{i,AC,R}$, $\delta _{i,AC,D})'\sim N(0,\boldsymbol {\varDelta }_{(4\times 4)})$. Assuming equal heterogeneities between treatment comparisons and equal correlations between random effects of different comparisons and different outcomes, i.e. corr $(\delta _{i,AB,R},\delta _{i,AC,D})={\mathrm {corr}}(\delta _{i,AB,D}, \delta _{i,AC,R})$, we show in Section 2 of supplementary material available at *Biostatistics* online that the $\boldsymbol {\varDelta }_{(4\times 4)}$ matrix takes the following form:

(3.5) \begin{align}\label{eq3.5} \boldsymbol{\varDelta}_{(4\times 4)} =\tau_{R}^{2} \left(\begin{matrix} 1 & 0 & 1/2 & 0 \\ 0 & 0 & 0 & 0 \\ 1/2 & 0 & 1 & 0 \\ 0 & 0 & 0 & 0 \\ \end{matrix}\right)+\tau_{D}^{2} \left(\begin{matrix} 0 & 0 & 0 & 0 \\ 0 & 1 & 0 & 1/2 \\ 0 & 0 & 0 & 0 \\ 0 & 1/2 & 0 & 1 \\ \end{matrix}\right)+\rho^{\tau}\tau_{R} \tau_{D} \left(\begin{matrix} 0 & 1 & 0 & 1/2 \\ 1 & 0 & 1/2 & 0 \\ 0 & 1/2 & 0 & 1 \\ 1/2 & 0 & 1 & 0 \\ \end{matrix}\right). \end{align}

When a considerable amount of data is available and the network is very dense (i.e. many studies connecting pairs of interventions) then the assumptions we used to reduce the number of model parameters might not be necessary, e.g. if there are at least three studies per comparison, then different heterogeneity variances can be used. However, real-life networks of interventions tend to be poorly connected and the median number of studies per comparison has been found to be low, equal to two studies (Nikolakopoulou *and others*, 2014). In Section 2 of supplementary material available at *Biostatistics* online, we present how $\boldsymbol {\varDelta }$ is modeled when correlations between different treatments and different outcomes are not equal. Note that the variance–covariance matrix as defined above is always positive definite. This matrix contains three parameters that need to be estimated: the heterogeneity variance $\tau _{R}^{2}$ for outcome $R$, the heterogeneity variance $\tau _{D}^{2}$ for outcome $D$, and the between-studies correlation coefficient $\rho ^{\tau }$, the same three parameters as in (3.2), assumed the same for each treatment comparison.

The random errors are also assumed to follow a multivariate normal distribution. For a three-arm study $i$ that compares treatments $A$, $B$ and $C$ for response ($R)$ and dropout ($D$), we assume $(\varepsilon _{i,AB,R},\varepsilon _{i,AB,D},\varepsilon _{i,AC,R}$, $\varepsilon _{i,AC,D})'\sim N(0,\boldsymbol {\varSigma }_{i})$. The variance–covariance matrix $\boldsymbol {\varSigma }_{i}$ is:

(3.6) \begin{equation*}\label{eq3.6} \boldsymbol{\varSigma}_{i} =\left(\begin{matrix} \sigma_{i,AB,R}^{2} & \cdot & \cdot & \cdot \\ \rho_{i,AB_{R} AB_{D} } \sigma_{i,AB,R} \sigma_{i,{\mathrm{A}}{\rm B},D} & \sigma_{i,AB,D}^{2} & \cdot & \cdot \\ \kappa_{i,AB_{R} AC_{R}} & \rho_{i,AC_{R} AB_{D} } \sigma _{i,AB,D} \sigma_{i,AC,D} & \sigma_{i,AC,R}^{2} & \cdot \\ \rho_{i,AB_{R} AC_{D} } \sigma_{i,AB,R} \sigma_{i,AC,D} & \kappa_{i,AB_{D} AC_{D}} & \rho_{i,AC_{R} AC_{D} } \sigma _{i,AC,R} \sigma_{i,AC,D} &\sigma_{i,AC,D}^{2} \\ \end{matrix}\right)\!. \end{equation*}

The $\sigma $ and $\kappa $ coefficients in $\boldsymbol {\varSigma }_{i}$ can be readily estimated if arm-level data are available. In the acute mania example, the variance of the $\log \,OR$ of the $AB$ comparison for response ($R)$ can be estimated as $\hat {\sigma }_{i,AB,R}^{2}=1/e_{i,A,R}+1/f_{i,A,R}+1/e_{i,B,R}+1/f_{i,B,R}$ and also $\hat {\kappa }_{i,{AB_{R}AC}_{R}}=1/e_{i,A,R}+1/f_{i,A,R}$ given the number of events ($e_{i,A,R}$, $e_{i,B,R})$ and failures ($f_{i,A,R}$, $f_{i,B,R})$ for each arm. In what follows, we present a method for dealing with the remaining correlation terms within $\boldsymbol {\varSigma }_{i}$. We start by assuming that there are two different types of within-study correlation coefficient for every study $i$: $\rho _{i}^{\ast }$ that correlates relative treatment effects of different outcomes for the same treatment comparison and enters the variance–covariance matrices for both two- and three-arm studies, and $\rho _{i}^{\ast \ast }$ that correlates relative treatment effects for different comparisons and different outcomes within the same study and enters only the $(4\times 4)$ matrices of the three-arm studies. This means that:

\[ \rho_{i,AB_{R} AB_{D} } =\rho_{i,AC_{R} AC_{D} } \equiv \rho_{i}^{\ast }, \quad\rho_{i,AC_{R} AB_{D} } =\rho_{i,AB_{R} AC_{D} }\equiv \rho_{i}^{\ast \ast}\quad (\hbox{Assumption}~1) \]

The within-study variance–covariance matrix for a two-arm study $i$ comparing treatments $A$ and $B$ for two outcomes is:

(3.7) \begin{equation*}\label{eq3.7} \boldsymbol{\varSigma}_{i} =\left(\begin{matrix} \sigma_{i,AB,R}^{2} & \rho_{i}^{\ast} \sigma_{i,AB,R} \sigma _{i,AB,D} \\ \rho_{i}^{\ast } \sigma_{i,AB,R} \sigma_{i,AB,D} & \sigma _{i,AB,D}^{2} \end{matrix}\right)\!. \end{equation*}

For a three-arm study comparing treatments $A$, $B$, and $C$ for two outcomes, the matrix of (3.6) becomes:

\[ \boldsymbol{\varSigma}_{i} =\left(\begin{matrix} \sigma_{i,AB,R}^{2} & \cdot & \cdot & \cdot \\ \rho_{i}^{\ast } \sigma_{i,AB,R} \sigma_{i,AB,D} &\sigma _{i,AB,D}^{2} & \cdot & \cdot \\ \kappa_{i,AB_{R} AC_{R}} &\rho_{i}^{\ast \ast } \sigma _{i,AB,D} \sigma_{i,AC,R} & \sigma_{i,AC,R}^{2} & \cdot \\ \rho_{i}^{\ast \ast } \sigma_{i,AB,R} \sigma_{i,AC,D} &\kappa_{i,AB_{D} AC_{D}} &\rho_{i}^{\ast } \sigma_{i,AC,R} \sigma_{i,AC,D} &\sigma_{i,AC,D}^{2} \\ \end{matrix}\right)\!. \]

It is very often the case that study arms are balanced in numbers of patients randomized. Then, for treatments that are not very different in efficacy and dropout (e.g. drugs from the same class) we can assume that

\[ \sigma_{i,BC,R}=\sigma_{i,AB,R}=\sigma_{i,AC,R}\quad \hbox{and }\sigma _{i,BC,D}=\sigma_{i,BA,D}=\sigma_{i,AC,D}\quad (\hbox{Assumption } 2) \]

This assumption will not be reasonable if trials are imbalanced or compare very different treatments. Insight on the validity of this assumption can be obtained from the data after scanning for important differences among the estimated variances across studies. If we choose to employ this assumption, the model is considerably simplified as it implies that $\rho _{i}^{\ast \ast }=1/2\rho _{i}^{\ast }$ (see Section 3 of supplementary material available at *Biostatistics* online). Consequently, an estimate of the variance–covariance matrix for the three-arm study $i$ after Assumptions 1 and 2 is as follows:

(3.8) \begin{align} \hat{\boldsymbol{\varSigma}}_{i} &=\left(\begin{matrix} \hat{\sigma }_{i,AB,R}^{2} & \cdot & \cdot & \cdot \\ 0 & \hat{\sigma}_{i,AB,D}^{2} & \cdot & \cdot \\ \hat{\kappa}_{i,AB_{R} AC_{R}} & 0 & \hat{\sigma}_{i,AC,R}^{2} & \cdot \\ 0 & \hat{\kappa}_{i,AB_{D} AC_{D}} & 0 &\hat{\sigma}_{i,AC,D}^{2} \\ \end{matrix}\right)\notag\\ &\quad+\rho_{i}^{\ast } \left(\begin{matrix} 0 & \cdot & \cdot & \cdot \\ \hat{\sigma}_{i,AB,R} \hat{\sigma}_{i,AB,D} & 0 & \cdot & \cdot \\ 0 & \dfrac{\hat{\sigma}_{i,AB,D} \hat{\sigma}_{i,AC,R}}{2} & 0 & \cdot \\ \dfrac{\hat{\sigma}_{i,AB,R} \hat{\sigma}_{i,AC,D} }{2} & 0 & \hat{\sigma }_{i,AC,R} \hat{\sigma}_{i,AC,D} & 0 \\ \end{matrix}\right)\equiv \hat{\boldsymbol{\varSigma}}_{i,1} +\rho_{i} \,\boldsymbol{\varSigma}_{i,2}\label{eq3.8} \end{align}

In the last line, we have renamed $\rho _{i}^{\ast }$ to $\rho _{i}$, in order to simplify notation and to highlight that the correlation coefficient is equivalent to the one presented in (3.3). It is important to note that Assumption 2 does not mean that we force all study variances to be equal: the diagonal elements of $\hat {\boldsymbol {\varSigma }}_{\boldsymbol{i}}$ are distinct and are estimated from the studies. We employ this assumption only for the offdiagonal elements of the variance–covariance matrix so that all correlations are functions of a single parameter $\rho _{i}$. Consequently, all elements of $\hat {\boldsymbol {\varSigma } }_{\boldsymbol{i},\textbf {1}}$ and $\hat {\boldsymbol {\varSigma }}_{\boldsymbol{i},\textbf {2}}$ in (3.8) can be estimated when arm-level data are available. The assumption of equal variances within a multi-arm study can be omitted, if it is deemed inappropriate. In Section 3 of supplementary material available at *Biostatistics* online, we present the most general form of the variance–covariance matrix for different variances and compute general relations between the correlation coefficients it contains. This, however, results in a rather complicated structure for the $\boldsymbol {\varSigma }_{\boldsymbol{i}}$ matrix and we will not consider it in this paper.

To summarize, we have expressed all within-study variance–covariance matrices utilizing a set of correlation coefficients $\rho _{i}$, one for every study $i$, that measure the correlation between the relative treatment effects of the two outcomes $R$ and $D$ for the same treatment comparison. These coefficients might be available in study reports or can be deducted from empirical evidence and expert opinion (Efthimiou *and others*, 2014; Riley, 2009). If IPD are available then the correlation coefficient can be estimated (Bujkiewicz *and others*, 2013). A joint NMA of the two outcomes can be performed within a Bayesian framework after assigning prior distributions to the $\rho _{i}$. These priors can be either uninformative or can be defined after consulting with clinicians (Wei and Higgins, 2013b). We have a number of options on how to model these coefficients. The simplest one is to assume $\rho _{i}=\rho $, common correlation for all studies. We could alternatively assume correlation coefficients across studies to share a common distribution. Another choice would be to have different $\rho _{i}$'s for different group of studies. For example, we could assume a coefficient $\rho _{{\mathrm {Act}}-{\mathrm { Pl}}}$ for placebo-controlled studies, and another $\rho _{{\mathrm {Act}}-{\mathrm { Act}}}$ for head-to-head studies that compare only active treatments; this would be based on the assumption that the two relative effect measures are differently correlated when one of the treatments compared is the placebo.

One technical implication that comes up is that the positive definiteness of the within-study variance–covariance matrix is not guaranteed for three-arm studies. The estimated matrix $\hat {\boldsymbol {\varSigma }}_{\boldsymbol{i}}$ for the random errors in (3.8) is not always positive definite, as it depends on the data and on an arbitrary parameter $\rho _{i}$. One way to overcome this problem is to compute the four eigenvalues $\lambda _{i,j}$ of $\hat {\boldsymbol {\varSigma }}_{\boldsymbol{i}}$ for every study $i$, with $j=1,2,3$, and 4, and truncate them to zero, replacing $\hat {\boldsymbol {\varSigma }}_{\boldsymbol{i}}=\sum _j \max (0,\lambda _{i,j})\boldsymbol{v}_{\boldsymbol{i},\boldsymbol{j}}\boldsymbol{v}_{\boldsymbol{i},\boldsymbol{j}}^{'}$, with $\boldsymbol{v}_{\boldsymbol{i},\boldsymbol{j}}$ the corresponding eigenvectors as in Jackson *and others* (2010). This, however, might be difficult to implement, particularly if a Bayesian software is used. Here, we propose a different way of dealing with this problem: we can truncate the correlation coefficient for every study so that the positive definiteness of the variance–covariance matrix is ensured. If for example we assume a uniform $(-1,1)$ prior distribution for each $\rho _{i}$, we must truncate: $\rho _{i}\sim Unif\, (-1,1)I(l_{i},u_{i})$. The limits $l_{i}$ and $u_{i}$ are the lowest and highest values of $\rho _{i}$ that lead to a positive-definite matrix. That means that we need to compute those values for all three-arm studies: it can be easily achieved by checking the corresponding eigenvalues of the variance–covariance matrix, as a positive-definite matrix has only positive eigenvalues. In Section 4 of supplementary material available at *Biostatistics* online, we provide a program in R software that computes the limits $l_{i}$ and $u_{i}$ for every three-arm study. Wei and Higgins discuss other approaches to ensure positive-definite matrices including Cholesky paramaterization and spherical decomposition (Wei and Higgins, 2013a).

#### 3.2.2 Model 2: Extending the alternative MOMA model

In this section, we discuss a second method for performing an MONMA, by extending Riley's *and others* alternative model (Riley *and others*, 2008). The model described in Section 3.1 is $\boldsymbol{Y}=\boldsymbol{X}\boldsymbol {\beta } +\boldsymbol {\eta }$, with $\boldsymbol {\eta }\sim N(0,\boldsymbol {\Omega })$, where, as in the case of pairwise meta-analysis the matrix $\boldsymbol {\Omega }$ is block diagonal. For a two-arm study, the variance–covariance matrix is as given in (3.4). As we show in Section 5 of supplementary material available at *Biostatistics* online, if we are willing to employ Assumption 2 for a three-arm study $i$ comparing treatments $A$, $B\,$and $C$ for two outcomes, then its variance–covariance matrix $\boldsymbol {\Omega }_{\boldsymbol{i}}$ is given by:

(3.9) \begin{equation*}\label{eq3.9} \Omega_{i} =\left(\begin{matrix} \zeta_{i,AB,R} & \cdot & \cdot & \cdot \\ \rho_{i}^{h} \sqrt{\zeta_{i,AB,R}\zeta_{i,AB,D}} &\zeta _{i,AB,D} & \cdot & \cdot \\ \dfrac{1}{2}\sqrt{\zeta_{i,AB,R} \zeta_{i,AC,R}} &\dfrac{\rho_{i}^{h}}{2}\sqrt{\zeta_{i,AB,D}\zeta_{i,AC,R}} & \zeta _{i,AC,R} & \cdot \\ \dfrac{\rho_{i}^{h}}{2}\sqrt{\zeta_{i,AB,R} \zeta_{i,AC,D}} & \dfrac{1}{2}\sqrt {\zeta_{i,AB,D} \zeta_{i,AC,D} } &\rho _{i}^{h} \sqrt {\zeta_{i,AC,R} \zeta_{i,AC,D} } &\zeta _{i,AC,D} \end{matrix}\right). \end{equation*}

Here we have defined $\zeta _{i,AB,R}=\sigma _{i,AB,R}^{2}+\psi _{R}^{2}$, $\zeta _{i,AB,D}=\sigma _{i,AB,D}^{2}+\psi _{D}^{2}$, etc. Equation (3.9) extends the model presented by Riley *and others* for three-arm studies with two outcomes. The $\sigma $ parameters can again be estimated from the data as the standard errors of the effect sizes, and assuming a common correlation coefficient across studies there are three parameters left to estimate: $\psi _{R}$, $\psi _{D}$, and $\rho ^{h}$. One of the advantages of this approach is that the variance-covariance matrix is always positive-definite, so a multivariate meta-analysis can be readily performed without further complications. As described in the previous section, the equal variance assumption (Assumption 2) can be omitted if the studies are imbalanced or the treatments have significant differences in the measured effects, leading, however, to a much more complicated $\boldsymbol {\varOmega }_{\boldsymbol{i}}$ variance-covariance matrix.

## 4. Application to acute mania dataset: NMA for response and dropout

### 4.1 Model fit and analysis plan

We fit both models in a Bayesian framework using the OpenBUGS software. Prior distributions must be assigned to all model parameters. The parameters $\tau _{R}$, $\tau _{D}$ of the first model and $\psi _{R}$, $\psi _{D}$ of the second can be assigned minimally informative prior distributions. If there is no prior information on the correlation of the outcomes, an uninformative $U(-1,1)$ prior can be used on all correlation coefficients. If external information is available on these coefficients, e.g. elicited from experts in the field, it can be used to inform $\rho $ or $\rho ^{h}$. In our example, the correlation between response and dropout rate is expected to be negative so we assigned appropriate negative priors to parameters $\rho _{i}$ (the within-study correlations between outcomes, assumed equal across studies), $\rho ^{\tau }$ (the between-study correlation in outcomes), and $\rho ^{h}$ (the overall correlation). However, the robustness of conclusions to this assumption could be checked if desired. In order to rank the treatments with respect to the response and the dropout rate, we computed the surface under the cumulative ranking curve, SUCRA (Salanti *and others*, 2011), for each treatment and for each outcome. For treatment $A$, outcome $R$, SUCRA is defined as $\sum _{k=1}^{N_{T}-1} {\mathrm {cum}}_{k}^{A,R}/(N_{T}-1)$, with ${\mathrm {cum}}_{k}^{A,R}$ denoting the probability of $A$ ranking among the best $k$ treatments for outcome $R$. SUCRA values lie between 0 (when the treatment is certain to be the worst for the outcome) and 1 (when the treatment is certain to be the best for the outcome). It is a transformation of the mean rank which takes uncertainty of estimation into account. All results pertain to 1 000 000 iterations and thinning of 100 after a 5000 burn-in period; the thinning was deemed necessary since a preliminary analysis showed a high auto-correlation in the chains. The code used is provided in Sections 6 and 7 of supplementary material available at *Biostatistics* online. We explored the following analysis scenarios:

I Univariate (independent) NMA of response and dropout rate separately, assuming $\tau _{R},\tau _{D}\sim U(0,1)$. This corresponds to setting all correlations equal to zero.
II MONMA following the approach of Section 3.2.1 with minimally informative priors for the heterogeneity parameters: $\rho ^{\tau }\sim U(-1,0)$, $\tau _{R},\tau _{D}\sim U(0,1)$, and (a) assuming a negative common $\rho _{i}=\rho $ with $\rho \sim U(-1,0)$; (b) assuming a strongly informative, negative, and common $\rho \sim U(-0.7,-0.5)$; (c) assuming a common fixed $\rho _{i}=\rho $ with $\rho =-0.7$; and (d) assuming two different within-studies correlation coefficients $\rho _{i}$: one for the studies comparing two active treatments, which we denote as $\rho _{{\mathrm {Act}}-{\mathrm { Act}}}$, and another for the studies comparing active treatments to placebo, $\rho _{{\mathrm {Act}}-{\mathrm { Pl}}}$. This distinction could be based on the assumption that the two relative treatment effects are differently correlated when one of the treatments compared is the placebo. For both parameters, we used a uniform negative, $U(-1,0)$, prior distribution.
III MONMA following the approach in Section 3.2.2, assuming a common correlation coefficient and the following prior distributions for the parameters of the model:
  \[ \rho^h\sim U(-1,0), \psi_R\sim U(0,1), \hbox{ and } \psi_D\sim U(0,1). \]

In order to evaluate our assumption of a negative correlation coefficient within and across studies we fitted MONMA model following the approach of Section 3.2.1 with $\rho _{i}=\rho $ with $\rho \sim U(-1,1)$ and $\rho ^{\tau }\sim U(-1,1)$.

### 4.2 Results

The median posterior values for $\rho $ and $\rho ^\tau $ when non-informative $U(-1,1)$ priors are used were $-$0.33 and $-$0.84 with 95% credible intervals [$-$0.66;0.14] and [$-$0.99;$-$0.38], respectively. These values corroborate our prior belief of a negative association between dropout and efficacy. In Table 1, we present the mean posterior estimates and 95% credible intervals for the parameters in each model. An interesting observation is that the heterogeneity variances $\tau _R^2$ and $\tau _D^2$ are invariant across the different models. This may be due to the large number of studies available in this meta-analysis. The mean estimates for the correlation coefficients are well below zero (e.g. the between-study correlation ranges from $-$0.56 for scenario II.c up to $-$0.82 for scenario II.a). The posterior median value for the overall correlation $\rho ^h$ in model III is $-$0.51 (95% credible intervals [$-$0.68;$-$0.29]), a value lying between the estimates of the two correlation coefficients for the multivariate model II.a ($-$0.34 for $\rho $ and $-$0.82 for $\rho ^\tau $). This is reasonable since $\rho ^h$ is an overall correlation coefficient that amalgamates the within- and between-studies correlations measured by $\rho $ and $\rho ^\tau $.

**Table 1.** Median posterior estimates and 95% credible intervals for the heterogeneity variance and correlation parameters in MONMA models.

Model	$\tau _{R}^{2}$	$\tau _{D}^{2}$	$\rho $	$\rho ^{\tau }$
I	0.08 [0.02; 0.17]	0.13 [0.06; 0.24]	—	—
II.a	0.07 [0.02; 0.16]	0.13 [0.06; 0.24]	$-0.34$ [$-$0.66; $-$0.04]	$-0.82$ [$-$0.99; $-$0.38]
II.b	0.07 [0.02; 0.15]	0.13 [0.06; 0.23]	$-0.56$ [$-$0.68; $-$0.50]	$-0.68$ [$-$0.93; $-$0.23]
II.c	0.07 [0.02; 0.16]	0.13 [0.06; 0.24]	—	$-0.56$ [$-$0.83; $-$0.12]
II.d	0.08 [0.02; 0.16]	0.13 [0.06; 0.23]	$\rho _{{\mathrm {Act}}-{\mathrm { Act}}}$: $-$0.31 [$-$0.71; $-$0.02]	$-0.80$ [$-$0.99; $-$0.33]
			$\rho _{{\mathrm {Act}}-{\mathrm { Pl}}}$: $-$0.39 [$-$0.77; $-$0.04]	
	$\psi _{R}^{2}$	$\psi _{D}^{2}$	$\rho ^{h}$	
III	0.07 [0.02;0.16]	0.12 [0.04;0.22]	$-0.51$ [$-$0.68; $-$0.29]	

**Fig. 1.**
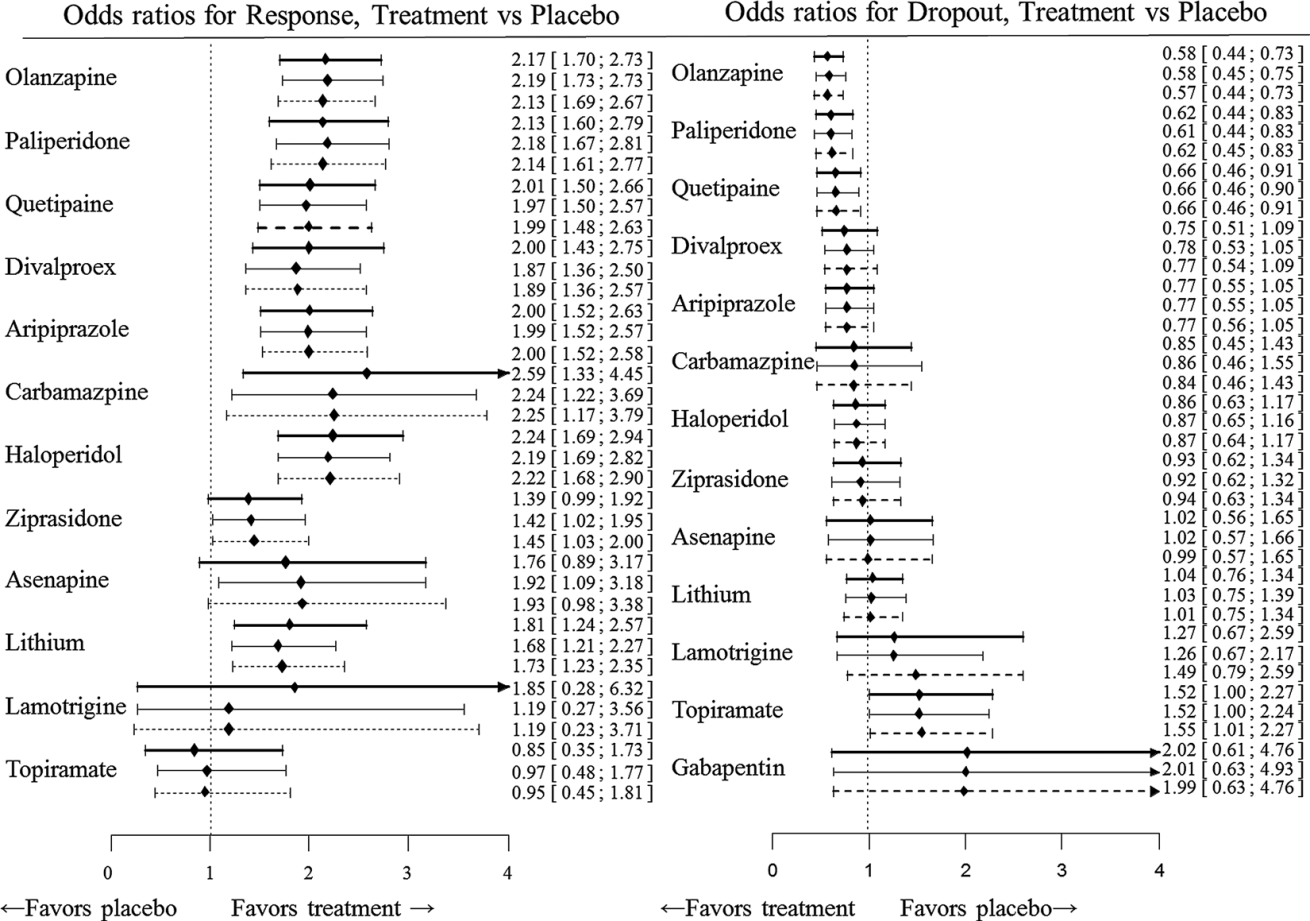
Summary odds ratios for response and dropout, for active treatment versus placebo. The thick lines correspond to model I (univariate model), the slim lines to model II.b (MONMA model assuming strong correlation coefficient $\rho \sim U(-0.7,-0.5)$) and the dashed lines to model III (alternative MONMA model assuming $\rho ^{\mathrm {h}}\sim U(-1,0)$.

In Figure 1, we present the summary odds ratios for both outcomes for each treatment versus placebo and for models I, II.b, and III. In Section 8 of supplementary material available at *Biostatistics* online, we present the results from fitting each model in detail. The multivariate approach has a minimal effect on the summary results for the dropout outcome compared with the univariate. That is expected (Riley *and others*, 2007a) since this outcome was reported in all studies except one, and thus inferences do not gain much through the joint analysis in terms of the posterior estimates and precision for this outcome. In contrast, the posterior summary ORs for the response to treatment outcome have considerable gain in precision when we use a multivariate rather than univariate model. This gain arises because 28% of the studies did not report on response, and thus the multivariate models additionally borrow strength from the correlated dropout outcome in these studies (Riley *and others*, 2007a). The gain in precision is larger as within-study correlation coefficient moves away from zero; the decrease in the width of the confidence intervals of the ORs compared with the results from the univariate approach is on average 8.4% for analysis II.a, 12% for II.b, 12.1% for II.c, 8.2% for II.d, and 10.8% for model III. Note that apart from differences in precision gain there are small changes in the point estimates for most odds ratios among the MONMA models (see Section 8 of supplementary material available at *Biostatistics* online).

**Fig. 2.**
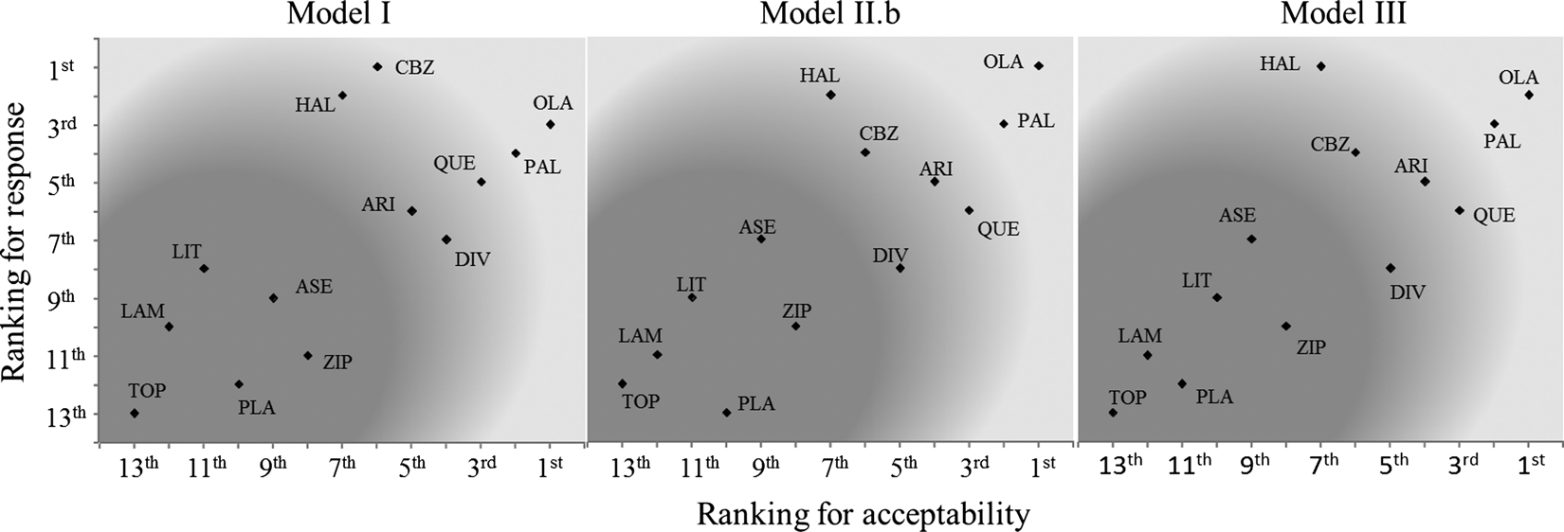
Ranking of antimanic drugs for response and acceptability. Treatments located in the darker (brighter) areas of the plots have the lowest (highest) rankings. ARI, aripiprazole; ASE, asenapine; CBZ, carbamazepine; VAL, divalproex; HAL, haloperidol; LAM, lamotrigine; LIT, lithium; OLZ, olanzapine; PBO, placebo; QTP, quetiapine; PAL, paliperidone; TOP, topiramate; ZIP, ziprasidone.

In Figure 2, we present the relative ranking of treatments for response and dropout, for models I, II.b and III, based on the SUCRA value for each outcome. Treatments near the upper right corner are the best when both acceptability and efficacy outcomes are considered jointly important; those near the bottom left corner (dark areas of the plots) are the worst. Note that Gabapentin is not present in the graph, since it was only reported for dropout. Regardless of the choice of model, OLA has the highest ranking across both outcomes jointly. However, the ranking of some other treatments is affected by the choice of multivariate rather than univariate, especially in regard the response outcome which (through correlation) is able to borrow strength from the more complete acceptability outcome. This use of additional information leads to (small) differences in the multivariate and univariate mean posterior estimates and precision of the summary ORs for response, and this has an impact on the relative ranking of the treatments for this outcome. For example, Carbamazepine ranks as the best treatment in terms of response with the univariate model but it falls to the fourth place when we consider a within-study correlation coefficient $\rho =-0.7$.

## 5. Discussion

We have developed two models for meta-analyzing evidence from multi-arm studies reporting multiple correlated outcomes in a network of interventions. Our models require minimum aggregated-level information, are independent of the mechanism that induces the correlations, and are applicable to any NMA with multiple continuous, dichotomous, or time-to-event outcomes; that is the majority of the NMA applications (Nikolakopoulou *and others*, 2014).

The set of models we present provides a unified way of handling multiple outcomes in the presence of multi-arm studies using only a handful of parameters. Choice between the two models may be informed by various factors. The first MONMA model accounts for within-study variances (sampling error), between-study variance (heterogeneity) as well as within and between-studies correlation. The second (alternative) model includes both within- and between-study variances, but uses a single correlation parameter $\rho ^{h}$. Thus, the second model can be viewed as an approximation of the first MONMA model, with the latter having a more detailed likelihood structure. The second model can be used in the common situation when within-study covariances (the $\kappa $ parameters in (3.6)) are not available from all studies or cannot be reliably obtained from external data or expert opinion. Ease of application is another consideration when choosing between the two models. The first model is more difficult to implement as it has a richer structure and investigators need to ensure the positive definiteness of the variance–covariance matrix.

Although NMA is an increasingly popular technique, few attempts have been made so far to combine it with multiple outcomes models. Our models are more general than those currently available in the literature. Welton *and others* (2008) presented an MONMA model applicable to NMA with two-arm studies only. Hong *and others* (2013) presented methods for synthesizing multiple outcomes for multiple competing treatments, but their approach completely ignores within-study correlations and we do not recommend it. Madan *and others* (2014) presented an approach for modeling multiple outcomes reported over multiple follow-up times; their models are applicable only for repeated measurements for a binary outcome and requires arm-level data.

Our models perform better than the univariate one in terms of precision; this gain, however, does not come without a cost. The complexity of the multivariate analysis is an important limitation, and the difficulty in implementing the models rises as the number of outcomes of interest or the number of arms of the studies in the network grows. When only a small number of studies do not report on all outcomes, the gain in precision can be trivial, rendering the use of multivariate methods redundant. The models are also limited by the assumptions we used to simplify the structure of the variance–covariance matrices; in supplementary material available at *Biostatistics* online, we offer guidance for the case the analyst is unwilling to employ these assumptions. The gain in power by joint modeling of correlated outcomes is sometimes too small to justify the increased modeling complexity (Trikalinos *and others*, 2013). Multivariate meta-analysis might provide more powerful results when several studies provide only one of the outcomes and in the presence of selective outcome reporting (Copas *and others*, 2014; Kirkham *and others*, 2012). We recommend to consider both multivariate and univariate approaches, to ascertain if clinical conclusions about the ranking of treatments for each outcome remain consistent under different model assumptions.

Despite their limitations the two presented models are to our knowledge the first attempts for meta-analyzing data from networks of interventions comprising multi-arm studies that report on multiple correlated outcomes. Efthimiou *and others* (2014) have developed a framework that utilizes expert clinical opinion about quantities easily understood by clinicians (such as proportions) to impute unreported correlation parameters. Their method is applicable only for binary outcomes measured with odds ratios. In the present approach, we provide two general models for all types of outcomes assuming that the within-study correlations are known or directly informed by external evidence (model 1) or completely unknown (model 2). Finally since MONMA is a new, largely unexplored area, there are still many open areas for research. A possible extension would be to include IPD, either exclusively or in a combination with aggregated data. Furthermore, our models could be implemented in popular statistical software making MONMA more easily accessible to review authors.

## Supplementary Material

Supplementary material is available at http://biostatistics.oxfordjournals.org

## Funding

This work was supported by the European Research Council (IMMA 260559 to Orestis Efthimiou, Dimitris Mavridis, and Georgia Salanti) and the MRC Methodology Research Programme (MR/J013595/1 to Richard D. Riley).

## Supplementary Material

Supplementary Data
