# Supplementary material for: Joint synthesis of multiple correlated outcomes in networks of interventions
Source: Biostatistics. 2014 Jul 2;16(1):84–97. doi: 10.1093/biostatistics/kxu030 (PMC4481542; doi:10.1093/biostatistics/kxu030)
Supplement: Supplementary Data [file supp_16_1_84__index.html]

Joint synthesis of multiple correlated outcomes in networks of interventions — Joint synthesis of multiple correlated outcomes in networks of interventions — Joint synthesis of multiple correlated outcomes in networks of interventions — Supplementary Data 

# Joint synthesis of multiple correlated outcomes in networks of interventions

## Supplementary Data

Supplementary Data

**Files in this Supplementary Material:**

- Supplementary Data - Docx file
