# Supplementary material for: Joint synthesis of multiple correlated outcomes in networks of interventions
Source: Biostatistics. 2014 Jul 2;16(1):84–97. doi: 10.1093/biostatistics/kxu030 (PMC4481542; doi:10.1093/biostatistics/kxu030)
Supplement: Supplementary Data [file supp_kxu030_kxu030supp.docx]

**Supplementary material to “Joint synthesis of multiple correlated outcomes in networks of interventions”**

ORESTIS EFTHIMIOU

*Department of Hygiene and Epidemiology, University of Ioannina School of Medicine, Ioannina, Greece.*

DIMITRIS MAVRIDIS

*Department of Hygiene and Epidemiology, University of Ioannina School of Medicine, Ioannina, Greece.*

*Department of Primary Education, University of Ioannina, Ioannina, Greece.*

RICHARD D. RILEY

*School of Health and Population Sciences, University of Birmingham, Birmingham, UK*

ANDREA CIPRIANI

*Department of Psychiatry, University of Oxford, Oxford, UK.*

GEORGIA SALANTI

*Department of Hygiene and Epidemiology, University of Ioannina School of Medicine, Ioannina, Greece.*

gsalanti@cc.uoi.gr

# THE ACUTE MANIA DATASET

Table 1 gives the number of events ($e$) and non-events ($f$) for the (binary) response and dropout outcomes for all studies in the network. Eighteen studies did not report for response, one did not report for dropout.

***Table 1. The acute mania dataset. $\boldsymbol{e}_{\boldsymbol{Ri}}$ and $\boldsymbol{f}_{\boldsymbol{Ri}}$ give the number of events and non-events for treatment $\boldsymbol{i}$, ($\boldsymbol{i=}$1, 2 or 3).***

|  | **Response** | | | | | | **Dropout** | | | | | | **Treatments being compared** | | |
| --- | --- | --- | --- | --- | --- | --- | --- | --- | --- | --- | --- | --- | --- | --- | --- |
| **Study** | $\boldsymbol{e}_{\boldsymbol{R}\boldsymbol{1}}$ | $\boldsymbol{e}_{\boldsymbol{R}\boldsymbol{2}}$ | $\boldsymbol{e}_{\boldsymbol{R}\boldsymbol{3}}$ | $\boldsymbol{f}_{\boldsymbol{R}\boldsymbol{1}}$ | $\boldsymbol{f}_{\boldsymbol{R}\boldsymbol{2}}$ | $\boldsymbol{f}_{\boldsymbol{R}\boldsymbol{3}}$ | $\boldsymbol{e}_{\boldsymbol{D}\boldsymbol{1}}$ | $\boldsymbol{e}_{\boldsymbol{D}\boldsymbol{2}}$ | $\boldsymbol{e}_{\boldsymbol{D}\boldsymbol{3}}$ | $\boldsymbol{f}_{\boldsymbol{D}\boldsymbol{1}}$ | $\boldsymbol{f}_{\boldsymbol{D}\boldsymbol{2}}$ | $\boldsymbol{f}_{\boldsymbol{D}\boldsymbol{3}}$ | **Treatment 1** | **Treatment 2** | **Treatment 3** |
| 1 | 155 | 63 |  | 98 | 68 |  | 54 | 20 |  | 199 | 111 |  | Aripiprazole | Placebo |  |
| 2 | 72 | 42 |  | 65 | 93 |  | 62 | 65 |  | 75 | 70 |  | Aripiprazole | Placebo |  |
| 3 | 89 | 72 |  | 86 | 100 |  | 41 | 77 |  | 134 | 95 |  | Aripiprazole | Haloperidol |  |
| 4 | 49 | 23 |  | 81 | 109 |  | 76 | 104 |  | 54 | 28 |  | Aripiprazole | Placebo |  |
| 5 | 110 | 49 |  | 157 | 85 |  | 157 | 80 |  | 110 | 54 |  | Aripiprazole | Placebo |  |
| 6 | 29 | 44 |  | 71 | 47 |  | 51 | 35 |  | 49 | 56 |  | Placebo | Quetipaine |  |
| 7 | 48 | 59 |  | 57 | 47 |  | 43 | 35 |  | 62 | 71 |  | Placebo | Quetipaine |  |
| 8 | 46 | 60 |  | 31 | 18 |  | 15 | 5 |  | 62 | 73 |  | Lithium | Quetipaine |  |
| 9 | 53 | 82 |  | 108 | 73 |  | 45 | 44 |  | 116 | 111 |  | Placebo | Quetipaine |  |
| 10 | 23 | 65 |  | 47 | 75 |  | 39 | 66 |  | 31 | 74 |  | Placebo | Ziprasidone |  |
| 11 | 19 | 63 |  | 47 | 77 |  | 30 | 55 |  | 36 | 85 |  | Placebo | Ziprasidone |  |
| 12 | 48 | 50 |  | 55 | 52 |  | 29 | 32 |  | 74 | 70 |  | Placebo | Ziprasidone |  |
| 13 |  |  |  |  |  |  | 10 | 12 |  | 5 | 2 |  | Ziprasidone | Olanzapine |  |
| 14 | 129 | 271 |  | 93 | 187 |  | 43 | 129 |  | 179 | 329 |  | Placebo | Ziprasidone |  |
| 15 |  |  |  |  |  |  | 3 | 1 |  | 12 | 14 |  | Lithium | Olanzapine |  |
| 16 | 16 | 34 |  | 53 | 36 |  | 45 | 27 |  | 24 | 43 |  | Placebo | Olanzapine |  |
| 17 | 24 | 35 |  | 36 | 20 |  | 35 | 21 |  | 25 | 34 |  | Placebo | Olanzapine |  |
| 18 | 68 | 52 |  | 57 | 74 |  | 39 | 45 |  | 86 | 81 |  | Olanzapine | Divalproex |  |
| 19 | 51 | 149 |  | 64 | 80 |  | 82 | 160 |  | 33 | 69 |  | Placebo | Olanzapine |  |
| 20 | 158 | 167 |  | 61 | 67 |  | 78 | 68 |  | 141 | 166 |  | Haloperidol | Olanzapine |  |
| 21 | 39 | 37 |  | 21 | 21 |  | 18 | 15 |  | 42 | 43 |  | Placebo | Olanzapine |  |
| 22 | 51 | 105 |  | 94 | 41 |  | 43 | 16 |  | 102 | 130 |  | Placebo | Paliperidone |  |
| 23 | 30 | 40 |  | 45 | 35 |  | 40 | 27 |  | 36 | 48 |  | Placebo | Paliperidone |  |
| 24 | 29 | 55 |  | 96 | 79 |  | 73 | 59 |  | 52 | 75 |  | Placebo | Paliperidone |  |
| 25 | 80 | 72 |  | 85 | 92 |  | 35 | 54 |  | 130 | 110 |  | Olanzapine | Paliperidone |  |
| 26 | 12 | 9 |  | 1 | 5 |  |  |  |  |  |  |  | Lithium | Divalproex |  |
| 27 | 60 | 89 |  | 125 | 103 |  | 89 | 81 |  | 96 | 111 |  | Placebo | Divalproex |  |
| 28 | 2 | 9 |  | 20 | 11 |  | 8 | 8 |  | 14 | 12 |  | Placebo | Divalproex |  |
| 29 | 11 | 8 |  | 4 | 7 |  | 3 | 3 |  | 12 | 12 |  | Divalproex | Carbamazpine |  |
| 30 | 30 | 47 |  | 37 | 22 |  | 11 | 7 |  | 56 | 62 |  | Placebo | Divalproex |  |
| 31 |  |  |  |  |  |  | 15 | 16 |  | 1 | 1 |  | Haloperidol | Carbamazpine |  |
| 32 | 54 | 112 |  | 166 | 111 |  | 110 | 93 |  | 110 | 130 |  | Placebo | Carbamazpine |  |
| 33 | 9 | 8 |  | 6 | 7 |  | 3 | 2 |  | 12 | 13 |  | Lithium | Lamotrigine |  |
| 34 |  |  |  |  |  |  | 28 | 87 |  | 72 | 127 |  | Placebo | Topiramate |  |
| 35 |  |  |  |  |  |  | 28 | 48 |  | 78 | 61 |  | Placebo | Topiramate |  |
| 36 | 52 | 60 |  | 19 | 9 |  | 15 | 6 |  | 56 | 63 |  | Lithium | Olanzapine |  |
| 37 | 51 | 156 |  | 71 | 191 |  | 50 | 132 |  | 72 | 215 |  | Placebo | Paliperidone |  |
| 38 | 5 | 4 |  | 4 | 4 |  | 7 | 2 |  | 2 | 6 |  | Haloperidol | Carbamazpine |  |
| 39 |  |  |  |  |  |  | 4 | 2 |  | 3 | 5 |  | Lithium | Haloperidol |  |
| 40 |  |  |  |  |  |  | 1 | 4 |  | 9 | 6 |  | Haloperidol | Carbamazpine |  |
| 41 |  |  |  |  |  |  | 5 | 1 |  | 14 | 14 |  | Lithium | Carbamazpine |  |
| 42 | 5 | 3 |  | 15 | 17 |  | 2 | 3 |  | 18 | 17 |  | Lithium | Olanzapine |  |
| 43 |  |  |  |  |  |  | 64 | 122 |  | 14 | 25 |  | Placebo | Divalproex |  |
| 44 | 32 | 26 |  | 112 | 117 |  | 13 | 25 |  | 131 | 118 |  | Placebo | Topiramate |  |
| 45 |  |  |  |  |  |  | 21 | 29 |  | 38 | 30 |  | Placebo | Gabapentin |  |
| 46 |  |  |  |  |  |  | 10 | 14 |  | 17 | 11 |  | Lithium | Carbamazpine |  |
| 47 | 30 | 40 |  | 71 | 61 |  | 41 | 43 |  | 60 | 58 |  | Placebo | Olanzapine |  |
| 48 | 56 | 72 | 71 | 109 | 83 | 89 | 87 | 82 | 82 | 78 | 73 | 78 | Placebo | Aripiprazole | Lithium |
| 49 | 58 | 78 | 80 | 95 | 89 | 85 | 44 | 41 | 44 | 109 | 126 | 121 | Placebo | Aripiprazole | Haloperidol |
| 50 | 26 | 52 | 57 | 71 | 46 | 50 | 30 | 14 | 10 | 67 | 84 | 97 | Placebo | Lithium | Quetipaine |
| 51 | 35 | 55 | 43 | 66 | 44 | 59 | 40 | 22 | 36 | 61 | 77 | 66 | Placebo | Haloperidol | Quetipaine |
| 52 | 18 | 93 | 65 | 70 | 79 | 113 | 63 | 94 | 105 | 25 | 78 | 73 | Placebo | Haloperidol | Ziprasidone |
| 53 | 31 | 82 | 75 | 74 | 133 | 126 | 28 | 56 | 50 | 77 | 159 | 151 | Placebo | Olanzapine | Divalproex |
| 54 |  |  |  |  |  |  | 25 | 28 | 18 | 26 | 25 | 34 | Placebo | Haloperidol | Paliperidone |
| 55 |  |  |  |  |  |  | 1 | 3 | 2 | 14 | 12 | 13 | Lithium | Haloperidol | Paliperidone |
| 56 | 39 | 59 | 65 | 101 | 85 | 89 | 21 | 14 | 17 | 119 | 130 | 137 | Placebo | Haloperidol | Paliperidone |
| 57 | 26 | 94 | 78 | 79 | 96 | 116 | 41 | 38 | 72 | 64 | 152 | 122 | Placebo | Olanzapine | Asenapine |
| 58 |  |  |  |  |  |  | 41 | 44 | 61 | 57 | 161 | 124 | Placebo | Olanzapine | Asenapine |
| 59 | 18 | 18 | 35 | 56 | 18 | 34 | 47 | 22 | 33 | 27 | 14 | 36 | Placebo | Lithium | Divalproex |
| 60 |  |  |  |  |  |  | 34 | 20 | 32 | 61 | 16 | 53 | Placebo | Lithium | Lamotrigine |
| 61 |  |  |  |  |  |  | 31 | 21 | 37 | 46 | 57 | 37 | Placebo | Lithium | Lamotrigine |
| 62 |  |  |  |  |  |  | 29 | 29 | 66 | 82 | 84 | 154 | Placebo | Lithium | Topiramate |
| 63 |  |  |  |  |  |  | 14 | 20 | 15 | 98 | 94 | 101 | Placebo | Lithium | Topiramate |
| 64 | 36 | 94 | 106 | 69 | 99 | 89 | 41 | 41 | 40 | 64 | 152 | 155 | Placebo | Quetipaine | Paliperidone |
| 65 | 43 | 13 | 53 | 56 | 7 | 52 | 47 | 12 | 33 | 52 | 8 | 72 | Placebo | Haloperidol | Olanzapine |

Figure 1 presents the network structure for each of the outcomes. For the graph we used the networkplot command in STATA (Chaimani et al. 2013).

*Figure 1: Network of pharmacological treatments for acute mania for response (on the left) and dropout (on the right). The size of each node corresponds to the number of studies reporting the treatment.*


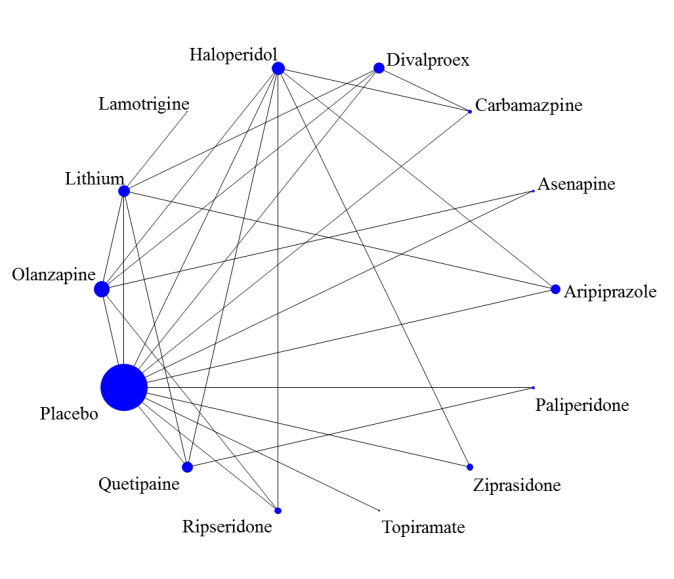

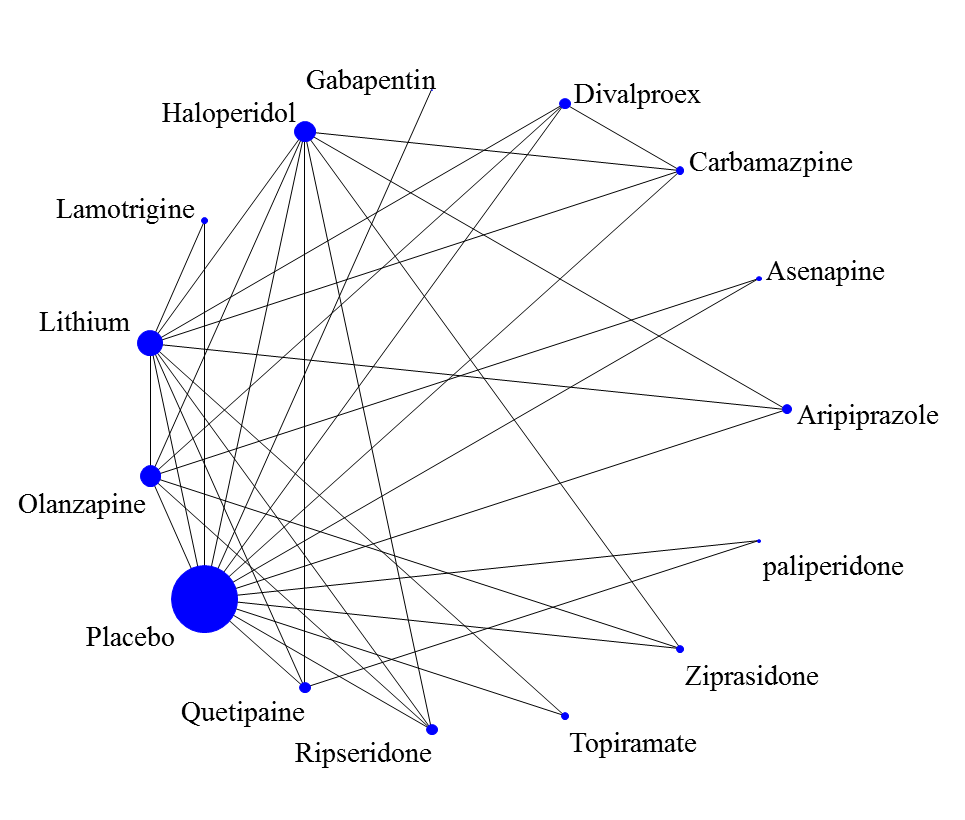


# THE VARIANCE-COVARIANCE MATRIX FOR HETEROGENEITY

For a three- arm study $i$ comparing treatments $A$, $B$ and $C$, the $(4\times4)$ variance-covariance matrix for the random effects is as follows:

$$\Delta_{(4\times4)}=\left(\begin{matrix} \tau_{R}^{2} & \rho^{\tau}\tau_{R}\tau_{D} & \tau_{R}^{2}/2 & \chi_{2}\tau_{R}\tau_{D} \\ \rho^{\tau}\tau_{R}\tau_{D} & \tau_{D}^{2} & \chi_{1}\tau_{R}\tau_{D} & \tau_{D}^{2}/2 \\ \tau_{R}^{2}/2 & \chi_{1}\tau_{R}\tau_{D} & \tau_{R}^{2} & \rho^{\tau}\tau_{R}\tau_{D} \\ \chi_{2}\tau_{R}\tau_{D} & \tau_{D}^{2}/2 & \rho^{\tau}\tau_{R}\tau_{D} & \tau_{D}^{2} \end{matrix}\right)$$

In the above matrix there are three different correlation coefficients $\rho^{\tau}$, $\chi_{1}$, $\chi_{2}$. Assuming similarity of the treatments being compared, a reasonable assumption to make is that $Corr\left( \delta_{i,AB,R},\delta_{i,AC,D} \right)=Cov\left( \delta_{i,AC,R},\delta_{i,AB,D} \right)=Corr\left( \delta_{i,BC,R},\delta_{i,BA,D} \right)=\ldots=\chi$. Thus in Equation we get $\chi_{1}=\chi_{2}=\chi$. If we choose a pair of random effects $\delta_{i,AB,D}$, $\delta_{i,AC,R}$ and compute the variance of their difference we get:

$$\begin{matrix} Var(\delta_{i,AC,D}-\delta_{i,AB,R})=Var(\delta_{i,AC,D})+Var(\delta_{i,AB,R})-2Cov(\delta_{i,AC,D},\delta_{AB,R})= \\ =\tau_{R}^{2}+\tau_{D}^{2}-2 \chi\tau_{R}\tau_{D} \end{matrix}$$

Assuming consistency we get:

$$\delta_{i,AC,D}-\delta_{i,AB,R}=\left(\delta_{i,BC,D}-\delta_{i,BA,D}\right)-\delta_{i,AB,R}=\delta_{i,BC,D}-\delta_{i,BA,D}+\delta_{i,BA,R}$$

By taking the variance of this equation it follows that:

$$\begin{matrix} Var(\delta_{i,AC,D}-\delta_{i,AB,R})=Var(\delta_{i,BC,D})+Var(\delta_{i,BA,D})+Var(\delta_{i,AB,R})-2Cov(\delta_{i,BC,D},\delta_{i,BA,D}) \\ +2Cov(\delta_{i,BC,D},\delta_{i,BA,R})-2Cov(\delta_{i,BA,D},\delta_{i,BA,R}) \end{matrix}$$

After using Equations and we get:

$$\tau_{D}^{2}+\tau_{R}^{2}-2 \chi\tau_{R}\tau_{D}=\tau_{D}^{2}+\tau_{D}^{2}+\tau_{R}^{2}-2\frac{\tau_{D}^{2}}{2}+2\chi\tau_{R}\tau_{D}-2\rho^{\tau}\tau_{R}\tau_{D}\Rightarrow$$

$$\chi=\frac{1}{2}\rho^{\tau}$$

Thus, the simplified form of the variance-covariance matrix $\Delta_{(4\times4)}$ is:

$$\Delta_{(4\times4)}=\left(\begin{matrix} \tau_{R}^{2} & \rho^{\tau}\tau_{R}\tau_{D} & \frac{\tau_{R}^{2}}{2} & \frac{\rho^{\tau}\tau_{R}\tau_{D}}{2} \\ \rho^{\tau}\tau_{R}\tau_{D} & \tau_{D}^{2} & \frac{\rho^{\tau}\tau_{R}\tau_{D}}{2} & \frac{\tau_{D}^{2}}{2} \\ \frac{\tau_{R}^{2}}{2} & \frac{\rho^{\tau}\tau_{R}\tau_{D}}{2} & \tau_{R}^{2} & \rho^{\tau}\tau_{R}\tau_{D} \\ \frac{\rho^{\tau}\tau_{R}\tau_{D}}{2} & \frac{\tau_{D}^{2}}{2} & \rho^{\tau}\tau_{R}\tau_{D} & \tau_{D}^{2} \end{matrix}\right)$$

We can decompose this matrix in terms of the three parameters that need to be estimated and three constant matrices:

$$\Delta_{(4\times4)}=\tau_{R}^{2}\left(\begin{matrix} 1 & 0 & \frac{1}{2} & 0 \\ 0 & 0 & 0 & 0 \\ \frac{1}{2} & 0 & 1 & 0 \\ 0 & 0 & 0 & 0 \end{matrix}\right)+\tau_{D}^{2}\left(\begin{matrix} 0 & 0 & 0 & 0 \\ 0 & 1 & 0 & \frac{1}{2} \\ 0 & 0 & 0 & 0 \\ 0 & \frac{1}{2} & 0 & 1 \end{matrix}\right)+\rho^{\tau}\tau_{R}\tau_{D}\left(\begin{matrix} 0 & 1 & 0 & \frac{1}{2} \\ 1 & 0 & \frac{1}{2} & 0 \\ 0 & \frac{1}{2} & 0 & 1 \\ \frac{1}{2} & 0 & 1 & 0 \end{matrix}\right)$$

$$\Rightarrow\Delta_{(4\times4)}=\tau_{R}^{2}\Delta_{1}+\tau_{D}^{2}\Delta_{2}+\rho^{\tau}\tau_{R}\tau_{D}\Delta_{3}$$

If we choose not to employ the $\chi_{1}=\chi_{2}=\chi$ assumption, then instead of we get that $\chi_{1}+\chi_{2}=\rho^{\tau}$ and Equation can be expressed in terms of two correlation parameters.

Note that the analysis in this Section holds for all type of outcomes. $R$ and $D$ may be binary (in which case we analyze the log odds ratios, log risk ratios or log hazard ratios), continuous (and we can use mean difference or standardized mean difference) or a mixture of binary and continuous, e.g. $R$ can be binary and $D$ continuous.

# THE VARIANCE-COVARIANCE MATRIX FOR RANDOM ERRORS

For a three-arm study $i$ that compares treatments $A$, $B$ and $C$ we assume that there are two different correlation coefficients, $\rho_{*}$ that correlates same comparisons-different outcomes, and $\rho_{**}$ for different comparisons different outcomes, i.e:

$\rho_{i,AB_{R}AB_{D}}=\rho_{i,AC_{R}AC_{D}}=\rho_{i,BC_{R}BC_{D}}\equiv\rho_{i}^{*}$, and $\rho_{i,AC_{R}AB_{D}}=\rho_{i,AB_{R}AC_{D}}=\rho_{i,BC_{R}BA_{D}}=\ldots\equiv\rho_{i}^{**}$

The variance-covariance matrix for the random errors in this study is the following:

$$\Sigma_{i}=\left(\begin{matrix} \sigma_{i,AB,R}^{2} & \rho_{i}^{*}\sigma_{i,AB,R}\sigma_{i,AB,D} & \kappa_{i,AB_{R}AC_{R}} & \rho_{i}^{**}\sigma_{i,AB,R}\sigma_{i,AC,D} \\ \rho_{i}^{*}\sigma_{i,AB,R}\sigma_{i,AB,D} & \sigma_{i,AB,D}^{2} & \rho_{i}^{**}\sigma_{i,AB,D}\sigma_{i,AC,R} & \kappa_{i,AB_{D}AC_{D}} \\ \kappa_{i,AB_{R}AC_{R}} & \rho_{i}^{**}\sigma_{i,AB,D}\sigma_{i,AC,R} & \sigma_{i,AC,R}^{2} & \rho_{i}^{*}\sigma_{i,AC,R}\sigma_{i,AC,D} \\ \rho_{i}^{**}\sigma_{i,AB,R}\sigma_{i,AC,D} & \kappa_{i,AB_{D}AC_{D}} & \rho_{i}^{*}\sigma_{i,AC,R}\sigma_{i,AC,D} & \sigma_{i,AC,D}^{2} \end{matrix}\right)$$

After assuming consistency we get:

$$y_{i,AC,R}-y_{i,BC,D}=y_{i,BC,R}-y_{i,BA,R}-y_{i,BC,D}$$

$y_{i,A,R}^{arm}$, $y_{i,B,R}^{arm}$ denote the observed relative treatment effects, measured in a suitable summary statistic relevant to the arm-specific binary or continuous outcome for study $i$, treatments $A$, $B$ and outcome $R$. For the contrast-level summary $y_{i,AB,R}$ (which may be log odds ratio, log risk ratio, log hazard ratio or mean difference) it holds that (Franchini et al. 2012):

$$Var(y_{i,AB,R})=Var(y_{i,A,R}^{arm})+Var(y_{i,B,R}^{arm})$$

For a multi-arm study $ABC$ it holds that:

$$Cov(y_{i,BC,R},y_{i,BA,R})=Var(y_{i,B,R}^{arm})$$

Using these two equations after taking the variance in both arms of Equation we get:

$$\begin{matrix} Var(y_{i,AC,R})+Var(y_{i,BC,D})-2\rho_{i}^{**}\sigma_{i,AC,R}\sigma_{i,BC,D}=Var(y_{i,BC,R})+Var(y_{i,BA,R})+ \\ +Var(y_{i,BC,D})-2Var(y_{i,B,R}^{arm})-2\rho_{i}^{*}\sigma_{i,BC,R}\sigma_{i,BC,D}+2\rho_{i}^{**}\sigma_{i,BA,R}\sigma_{i,BC,D} \end{matrix}$$

$$\begin{matrix} \Rightarrow Var(y_{i,A,R}^{arm})+Var(y_{i,C,R}^{arm})+Var(y_{i,B,D}^{arm})+Var(y_{i,C,D}^{arm})-2\rho_{i}^{**}\sigma_{i,AC,R}\sigma_{i,BC,D} \\ =Var(y_{i,B,R}^{arm})+Var(y_{i,C,R}^{arm})+Var(y_{i,B,R}^{arm})+Var(y_{i,A,R}^{arm})+Var(y_{i,B,D}^{arm})+Var(y_{i,C,D}^{arm}) \\ -2Var(y_{i,B,R}^{arm})-2\rho_{i}^{*}\sigma_{i,BC,R}\sigma_{i,BC,D}+2\rho_{i}^{**}\sigma_{i,BA,R}\sigma_{i,BC,D} \end{matrix}$$

This leads to:

$$\rho_{i}^{**}=\rho_{i}^{*}\frac{\sigma_{i,BC,R}}{\sigma_{i,BA,R}+\sigma_{i,AC,R}}$$

If we also assume that the standard deviations of different comparisons of the same outcome are equal within every study, i.e. $\sigma_{i,BC,R}=\sigma_{i,BA,R}=\sigma_{i,AC,R}$, we get that

$$\rho_{i}^{**}=\frac{1}{2}\rho_{i}^{*}$$

Note that in order for this to be a consistent result we must further assume that $\sigma_{i,BC,D}=\sigma_{i,BA,D}=\sigma_{i,AC,D}$.

Even though we have assumed equal variances to simplify the variance-covariance matrix of Equation , in the end of the day the $\sigma$ and $\kappa$ parameters are still left distinct and are estimated from the data. Equation is just used to minimize the number of correlation parameters needed for the matrix in Equation .

The two assumptions we used (equal correlations, equal variances) are a justified approximation when all treatments in each study are comparable and the arms are balanced. This, however, may not always be the case. We can repeat the whole analysis without making any assumptions of equality in either the correlation coefficients or the variances. By taking analogous relations to the one in Equation we get the following set of equations, after dropping the study index $i$ for simplicity:

$$\begin{matrix} \rho_{AC_{R}AC_{D}}\sigma_{AC_{R}}=\rho_{AB_{R}AC_{D}}\sigma_{AB_{R}}+\rho_{BC_{R}AC_{D}}\sigma_{BC_{R}} \\ \rho_{AC_{R}AC_{D}}\sigma_{AC_{D}}=\rho_{AC_{R}AB_{D}}\sigma_{AB_{D}}+\rho_{AC_{R}BC_{D}}\sigma_{BC_{D}} \\ \rho_{AB_{R}AB_{D}}\sigma_{AB_{R}}=\rho_{AC_{R}AB_{D}}\sigma_{AC_{R}}-\rho_{BC_{R}AB_{D}}\sigma_{BC_{R}} \\ \rho_{AB_{R}AB_{D}}\sigma_{AB_{D}}=\rho_{AB_{R}AC_{D}}\sigma_{AC_{D}}-\rho_{AB_{R}BC_{D}}\sigma_{BC_{D}} \\ \rho_{BC_{R}AB_{D}}\sigma_{BC_{R}}=\rho_{AC_{R}BC_{D}}\sigma_{AC_{R}}-\rho_{AB_{R}BC_{D}}\sigma_{AC_{R}} \end{matrix}$$

By cycling through the treatment and outcome indices we can produce more equations of this form, but it turns out they are linearly dependent to the ones above. Thus, out of the nine different correlation coefficients entering the five Equations only four of them are independent. This set of equations is the most general solution to the problem of finding the correlation coefficients in a three-arm study. The only assumption used to obtain them is the consistency of the network.

Depending on the nature of the problem one can now make extra assumptions to simplify these equations. If for example out of the three treatments $A, B$ and $C$ being compared in a study, $A$ is the placebo, while the other two are active treatments with similar results in both outcomes, it would be justifiable to assume $\rho_{{AC}_{R}{AB}_{D}}=\rho_{{AB}_{R}{AC}_{D}}=\rho^{**}$. If we also set $\rho_{{AB}_{R}{AB}_{D}}=\rho_{{AC}_{R}{AC}_{D}}=\rho_{{BC}_{R}{BC}_{D}}=\rho^{*}$, we find:

$$\rho^{**}=\frac{\sigma_{AB_{R}}\sigma_{AB_{D}}+\sigma_{AC_{R}}\sigma_{AC_{D}}-\sigma_{BC_{R}}\sigma_{BC_{D}}}{\sigma_{AB_{R}}\sigma_{AC_{D}}+\sigma_{AB_{D}}\sigma_{AC_{R}}}\rho^{*}$$

This equation allows a simplification of the variance-covariance matrix without the need of any assumption on the variances of the treatment effects. Also note that this equation further reduces to Equation by employing the equal variance assumption.

Finally, note that the analysis presented in this Section is not strictly valid for the case of standardized mean difference (SMD); this is because Equations and do not apply in this case. The exact formulas can be found in (Gleser and Olkin 2009), equations 19.18 and 19.19. However, for large sample sizes it holds that $Var(SMD_{i,AB,R})\approx\frac{1}{n_{i,A,R}}+\frac{1}{n_{i,B,R}}$ and $Cov(SMD_{i,AB,R},SMD_{i,AC,R})\approx\frac{1}{n_{i,A,R}}$. In this approximation Equations and hold and the analysis presented in this section is also applicable for the case when SMD is used for one or more of the outcomes.

# *ENSURING THE POSITIVE-DEFINITENESS OF THE VARIANCE-COVARIANCE MATRIX OF MODEL II*

The correlation coefficient parameter $\rho_{i}$ that enters the model described in Section 3.2.1 of the main paper needs to be truncated separately for each three-arm study in order to ensure the positive-definiteness of the variance-covariance matrix of Equation (8) of the main paper. The R program used to compute the (study-specific) upper limits $u_{i}$ for the correlation coefficient is the following:

rho=c(rep(0,N))

ff=function(r,m){

s=s1+r*s2

ss=eigen(s[m,,],only.values = TRUE)

mineg=min(ss[[1]])

mineg}

for(m in 1:N){

for(i in 1:100){

if (ff(0.01*i,m)*ff(0.01*i+0.01,m)<0){rho[m]=0.01*i}}}

rho

The program utilizes the fact that a positive-definite matrix has only positive eigenvalues. The inputs needed are the number $N$ of the three-arm studies and two arrays s1 and s2 which are $(N\times4\times4)$-dimensional and contain the $N$ in number $(4\times4)$-dimensional matrices $\boldsymbol{S}_{\boldsymbol{i,1}}$ and $\boldsymbol{S}_{\boldsymbol{i,2}}$ of Equation (8) of the paper, which are estimated from the data. A similar program can be used to compute the lower values, but the limits are symmetrical around zero. The results for the 18 three-arm studies of the acute mania dataset are given in Table 2.

*Table 2. Upper limit for the correlation coefficient in the three-arm studies*

| **Study** | $\boldsymbol{u}_{\boldsymbol{i}}$ |
| --- | --- |
| 48 | 0.99 |
| 49 | 0.96 |
| 50 | 0.96 |
| 51 | 0.98 |
| 52 | 0.54 |
| 53 | 0.65 |
| 54 | 0.81 |
| 55 | 0.86 |
| 56 | 0.99 |
| 57 | 0.68 |
| 58 | 0.84 |
| 59 | 0.95 |
| 60 | 0.78 |
| 61 | 0.80 |
| 62 | 0.83 |
| 63 | 0.82 |
| 64 | 0.75 |
| 65 | 0.85 |

# GENERALIZING THE ALTERNATIVE MODEL BY RILEY ET AL*.*

In a paper by Riley et al. (Riley, Thompson, and Abrams 2008) the authors propose a model for bivariate pairwise meta-analysis in which they allow for a single correlation coefficient to model all correlations; this hybrid coefficient incorporates both within and between-study correlation. For a two-arm study $i$ reporting on outcomes $R$ and $D$ a bivariate normal distribution is assumed:

$$\left(\begin{matrix} y_{i,R} \\ y_{i,D} \end{matrix}\right)\sim N\left(\left(\begin{matrix} \beta_{1} \\ \beta_{2} \end{matrix}\right),\text{Ω}_{i}\right)$$

With the variance-covariance matrix given by (assuming a common correlation coefficient $\rho^{h}$ for simplicity):

$$\text{Ω}_{i}=\left(\begin{matrix} \sigma_{i,AB,R}^{2}+\psi_{R}^{2} & \rho^{h}\sqrt{\left(\sigma_{i,AB,R}^{2}+\psi_{R}^{2}\right)\left(\sigma_{i,AB,D}^{2}+\psi_{D}^{2}\right)} \\ \rho^{h}\sqrt{\left(\sigma_{i,AB,R}^{2}+\psi_{R}^{2}\right)\left(\sigma_{i,AB,D}^{2}+\psi_{D}^{2}\right)} & \sigma_{i,AB,D}^{2}+\psi_{D}^{2} \end{matrix}\right)$$

The $\psi$ parameters model for the additional variation apart from the sampling error that enters due to heterogeneity. Here we show how to extend the model for the case of a network of interventions. We restrict to the case of networks with two-arm and three-arm studies only. For a two-arm study $i$ comparing treatments $A$ and $B$ for outcomes $R$ and $D$, the variance-covariance matrix is again of the form of Equation . For a three-arm study comparing $A$, $B$ and $C$ treatments the variance-covariance matrix of Equation can be generalized as follows:

$$\text{Ω}_{i}=\left(\begin{matrix} \zeta_{i,AB,R} & . & . & . \\ \rho^{h}\sqrt{\zeta_{i,AB,R}\zeta_{i,AB,D}} & \zeta_{i,AB,D} & . & . \\ \rho_{1}\sqrt{\zeta_{i,AB,R}\zeta_{i,AC,R}} & \rho_{2}\sqrt{\zeta_{i,AB,D}\zeta_{i,AC,R}} & \zeta_{i,AC,R} & . \\ \rho_{2}\sqrt{\zeta_{i,AB,R}\zeta_{i,AC,D}} & \rho_{3}\sqrt{\zeta_{i,AB,D}\zeta_{i,AC,D}} & \rho^{h}\sqrt{\zeta_{i,AC,R}\zeta_{i,AC,D}} & \zeta_{i,AC,D} \end{matrix}\right)$$

In the above we have set $\zeta_{i,AB,R}=\sigma_{i,AB,R}^{2}+\psi_{R}^{2}$ and $\zeta_{i,AB,D}=\sigma_{i,AB,D}^{2}+\psi_{D}^{2}$, similarly for the $AC$ comparison. In Equations and we have assumed that the correlation coefficient $\rho^{h}$ correlates treatment effects of the same treatment comparison but different outcomes (e.g. comparison $AB$ for outcomes $R$ and $D$), $\rho_{1}$ correlates different treatment comparisons of the $R$ outcome, $\rho_{2}$ correlates different outcomes of different comparison and $\rho_{3}$ correlates different comparisons of the $D$ outcome. In order to simplify this matrix we also assume that the variances of the contrast-level treatment effects for comparisons of the same outcome are equal within a study, irrespectively of the comparison being made:

$\sigma_{i,AB,R}^{2}=\sigma_{i,AC,R}^{2}=\sigma_{i,BC,R}^{2}$ and $\sigma_{i,AB,D}^{2}=\sigma_{i,AC,D}^{2}=\sigma_{i,BC,D}^{2}$.

This assumption, with the use of the consistency equations leads to $\rho_{1}=\rho_{3}=1/2$, as it is easy to prove. For example, consistency states that $y_{i,AB,R}=y_{i,AC,R}-y_{i,BC,R}$. By taking the variance on both sides we get:

$$\zeta_{i,AB,R}=\zeta_{i,AC,R}+\zeta_{i,BC,R}-2\rho_{1}\sqrt{\zeta_{i,AB,R}\zeta_{i,BC,R}}\Rightarrow\rho_{1}=1/2$$

In the above we use $\zeta_{i,AB,R}=\zeta_{i,AC,R}=\zeta_{i,BC,R}$, which holds by virtue of the equal variance assumption we make. A similar proof holds for $\rho_{3}$.

Also, as we proved in Equation , the consistency equations give:

$$Var(y_{i,AC,R})+Var(y_{i,BC,D})-2Cov(y_{i,AC,R},y_{i,BC,D})=Var(y_{i,BC,R})+Var(y_{i,BA,R})+$$

$$Var(y_{i,BC,D})-2Cov(y_{i,BC,R},y_{i,BA,R})-2Cov(y_{i,BC,R},y_{i,BC,D})+2Cov(y_{i,BA,R},y_{i,BC,D})$$

$$\Rightarrow\zeta_{i,R}+\zeta_{i,D}-2\rho_{2}\sqrt{\zeta_{i,R}\zeta_{i,D}}=$$

$$=\zeta_{i,R}+\zeta_{i,R}+\zeta_{i,D}-2\rho_{1}\sqrt{\zeta_{i,R}\zeta_{i,R}}-2\rho^{h}\sqrt{\zeta_{i,R}\zeta_{i,D}}+2\rho_{2}\sqrt{\zeta_{i,R}\zeta_{i,D}}$$

In the above we have set $\zeta_{i,AB,R}=\zeta_{i,AC,R}=\zeta_{i,BC,R}=\zeta_{i,R}$ and $\zeta_{i,AB,D}=\zeta_{i,AC,D}=\zeta_{i,BC,D}=\zeta_{i,D}$. By substituting $\rho_{1}=1/2$, and after some algebra we get:

$$\rho_{2}=\frac{1}{2}\rho^{h}$$

The variance-covariance matrix takes the following form:

$$\text{Ω}_{i}=\left(\begin{matrix} \zeta_{i,AB,R} & . & . & . \\ \rho^{h}\sqrt{\zeta_{i,AB,R}\zeta_{i,AB,D}} & \zeta_{i,AB,D} & . & . \\ \frac{1}{2}\sqrt{\zeta_{i,AB,R}\zeta_{i,AC,R}} & \frac{\rho^{h}}{2}\sqrt{\zeta_{i,AB,D}\zeta_{i,AC,R}} & \zeta_{i,AC,R} & . \\ \frac{\rho^{h}}{2}\sqrt{\zeta_{i,AB,R}\zeta_{i,AC,D}} & \frac{1}{2}\sqrt{\zeta_{i,AB,D}\zeta_{i,AC,D}} & \rho^{h}\sqrt{\zeta_{i,AC,R}\zeta_{i,AC,D}} & \zeta_{i,AC,D} \end{matrix}\right)$$

The $\sigma$ parameters entering the $\zeta$ can be estimated from the data. This variance-covariance matrix is always positive-definite.

# OPENBUGS CODE FOR MODEL II

The following program in OpenBUGS was used for fitting the acute mania network to the first model presented in the main paper, Section 3.2.1:

model{

*# this controls for studies with one outcome not reported by setting # the correlation equal to*

*# zero*

for (k in 1:(2*Ns-N2h))

{control[k]<-step(9999-varr[k])*step(9999-vard[k])}

*# two-arm studies*

for( k in 1:N2h){

s[k,1,1]<-varr[k]

s[k,2,2]<-vard[k]

s[k,1,2]<-control[k]*rhosigma*sqrt(varr[k]*vard[k])

s[k,2,1]<-control[k]*rhosigma*sqrt(varr[k]*vard[k])

prec2A[k,1:2,1:2]<-inverse(s[k,,])

y[(2*k-1):(2*k)]~dmnorm(theta[(2*k-1):(2*k)],prec2A[k,,])

for(i in 1:2){

for (j in 1:2){

D2[k,i,j]<-tau1.sq*t1[i,j]+tau2.sq*t2[i,j]+sqrt(tau1.sq*tau2.sq)*control[k]*(rhotau)*t3[i,j]}}

prec2B[k,1:2,1:2]<-inverse(D2[k,,])

theta[(2*k-1):2*k]~dmnorm(mean[(2*k-1):2*k],prec2B[k,,]) }

*# three-arm studies*

for (k in 1:(Ns-N2h)){

rhosigma1T[k]<-max(rhosigma,-ul[k])

rhosigmaT[k]<-min(rhosigma1T[k],ul[k])

for (i in 1:4){

for (j in 1:4){

S[k,i,j]<-sigma1[k,i,j]+control[k]*rhosigmaT[k]*sigma2[k,i,j]}}

prec3A[k,1:4,1:4]<-inverse(S[k,,])

y[2*N2h+4*k-3:2*N2h+4*k]~dmnorm(theta[2*N2h+4*k-3:2*N2h+4*k],prec3A[k,,])

for (i in 1:4){

for (j in 1:4){

D3[k,i,j]<-tau1.sq*delta1[i,j]+tau2.sq*delta2[i,j]+sqrt(tau1.sq*tau2.sq)*(control[k]*rhotau)*delta3[i,j]}}

prec4A[k,1:4,1:4]<-inverse(D3[k,,])

theta[2*N2h+4*k-3:2*N2h+4*k]~dmnorm(mean[2*N2h+4*k-3:2*N2h+4*k],prec4A[k,,])}

*# Parameterization of the means*

for(i in 1:N2h) {

mean[2*i-1] <- -dR[T2[i]] + dR[T1[i]]

mean[2*i] <- -dD[T2[i]]+ dD[T1[i]]}

for(i in 1:(Ns-N2h)) {

mean[2*N2h+4*i-3] <- -dR[T2[N2h+i]] + dR[T1[N2h+i]]

mean[2*N2h+4*i-2] <- -dD[T2[N2h+i]] + dD[T1[N2h+i]]

mean[2*N2h+4*i-1] <- -dR[T3[N2h+i]]+ dR[T1[N2h+i]]

mean[2*N2h+4*i] <- -dD[T3[N2h+i]] + dD[T1[N2h+i]] }

*# Priors*

for(k in 1:(ref-1)) {dR[k] ~ dnorm(0,.01)}

for(k in (ref+1):NT) {dR[k] ~ dnorm(0,.01)}

for(k in 1:(ref-1)) {dD[k] ~ dnorm(0,.01)}

for(k in (ref+1):NT) {dD[k] ~ dnorm(0,.01)}

tau1.sq<-tau1*tau1

tau1~dunif(0,1)

tau2.sq<-tau2*tau2

tau2~dunif(0,1)

rhosigma<-0

rhotau<-0

*# Estimated Effect Sizes*

dR[ref]<- 0

for (c in 1:(ref-1)) {Eff.refR[c]<- exp(dR[c] - dR[ref] )}

for (c in (ref+1):NT) {Eff.refR[c]<- exp(dR[c] - dR[ref] )}

for (c in 1:(NT-1)) {

for (k in (c+1):NT) {EffR[c,k] <- exp(dR[k] - dR[c])}}

dD[ref]<- 0

for (c in 1:(ref-1)) {Eff.refD[c]<- exp(dD[c] - dD[ref] )}

for (c in (ref+1):NT) { Eff.refD[c]<- exp(dD[c] - dD[ref] )}

for (c in 1:(NT-1)) {

for (k in (c+1):NT) {EffD[c,k] <- exp(dD[k] - dD[c])}}

*# SUCRA rankings*

*# Ranking of treatments for response. This part is customized for the acute mania dataset,*

*# where one of the treatments was not reported for response.*

for(k in 1:13) {ddR[k]<-dR[k]}

for(k in 1:13) {

orderR[k]<-14- rank(ddR[],k)

most.effectiveR[k]<-equals(orderR[k],1)

for(j in 1: 13) {

effectivenessR[k,j]<- equals(orderR[k],j)

cumeffectivenessR[k,j]<- sum(effectivenessR[k,1:j])}}

for(k in 1:13) {

SUCRAR[k]<- sum(cumeffectivenessR[k,1:(13-1)]) /(13-1)}

*#Ranking of treatments for dropout*

for(k in 1:NT) {

orderD[k]<- rank(dD[],k)

most.effectiveD[k]<-equals(orderD[k],1)

for(j in 1: NT) {

effectivenessD[k,j]<- equals(orderD[k],j)

cumeffectivenessD[k,j]<- sum(effectivenessD[k,1:j])}}

for(k in 1:NT) {

SUCRAD[k]<- sum(cumeffectivenessD[k,1:(NT-1)]) /(NT-1))}}

The inputs required for this program are the following:

N2h: the number of two-arm studies.

Ns: the total number of studies.

NT: the number of treatments.

ref: the treatment number for the reference treatment (e.g. placebo).

y: the $2(N2h+2Ns)$-dimensional vector of observed effects (two for every two-arm study, four for every three-arm). Odd positions correspond to $R$ comparison, even to $D$. Impute NA when in a study an outcome is missing.

varr: the $(N2h+2Ns$)-dimensional vector of the variance for every $R$ comparison (one for each odd position in y). For studies with missing data impute a large variance (10,000).

vard: the $(N2h+2Ns)$- dimensional vector of the variance for every $D$ comparison (one for each even position in y). For studies with missing data impute a large variance (10,000).

T1, T2, T3: these are $Ns$– dimensional vector of treatments for every study. T1 refers to the first treatment of every study (chosen arbitrarily), T2 to the second. For two arm-studies set T3 = 0.

t1, t2, t3: the $(2\times2)$ matrices needed for constructing the heterogeneity variance-covariance matrix for the two-arm studies, $\Delta_{(2\times2)}$ in Equation (2) of the paper:

t1 = structure(.Data=c(1, 0, 0, 0),.Dim=c( 2 , 2 ))

t2 = structure(.Data=c(0, 0, 0, 1),.Dim=c( 2 , 2 ))

t3 = structure(.Data=c(0, 1, 1, 0),.Dim=c( 2 , 2 ))

delta1, delta2, delta3: the $(4\times4)$ matrices needed for constructing the heterogeneity variance-covariance matrix $\Delta_{(4\times4)}$ for the three-arm studies, Equation (5) of the paper.

delta1 = structure(.Data=c(1,0,0.5 0,0,0,0,0,0.5,0,1,0,0,0,0,0),.Dim=c(4,4 ))

delta2 = structure(.Data=c(0,0,0,0,0,1,0,0.5,0,0,0,0,0,0.5,0,1),.Dim=c(4 ,4))

delta3=structure(.Data=c(0,1,0,0.5,1,0,0.5,0,0,0.5,0,1,0.5,0,1,0),.Dim=c(4,4))

sigma1, sigma2: the $(Ns-N2h)\times4\times4$ – dimensional arrays entering Equation (8) for every three-arm study, as computed from the data.

# OPENBUGS CODE FOR MODEL III

The following OpenBUGS code was used for analyzing the acute mania dataset using the second model presented in the main paper, Section 3.2.2 of the paper.

model{

*# this controls for studies with one outcome not reported by setting the correlation equal to*

*# zero*

for (k in 1:(2*Ns-N2h)){control[k]<-step(9999-varr[k])*step(9999-vard[k])}

*# two-arm studies*

for( i in 1:N2h){

s[i,1,1]<-varr[i]+psiR.sq

s[i,2,2]<-vard[i]+psiD.sq

s[i,1,2]<-control[i]*rho1*sqrt(varr[i]+psiR.sq)*sqrt(vard[i]+psiD.sq)

s[i,2,1]<-s[i,1,2]

prec2A[i,1:2,1:2]<-inverse(s[i,,])

y[(2*i-1):(2*i)]~dmnorm(mean[(2*i-1):(2*i)],prec2A[i,,])}

*# three-arm studies*

for (i in 1:(Ns-N2h)){

S[i,1,1]<- varr[N2h+2*i-1]+psiR.sq

S[i,2,2]<-vard[N2h+2*i-1]+psiD.sq

S[i,3,3]<- varr[N2h+2*i]+psiR.sq

S[i,4,4]<-vard[N2h+2*i]+psiD.sq

S[i,1,2]<- control[i]*rho1*sqrt(S[i,1,1])*sqrt(S[i,2,2])

S[i,2,1]<- S[i,1,2]

S[i,1,3]<- control[i]*sqrt(S[i,1,1])*sqrt(S[i,3,3])/2

S[i,3,1]<- S[i,1,3]

S[i,1,4]<- control[i]*rho1*sqrt(S[i,1,1])*sqrt(S[i,4,4])/2

S[i,4,1]<- S[i,1,4]

S[i,2,3]<- control[i]*rho1*sqrt(S[i,2,2])*sqrt(S[i,3,3])/2

S[i,3,2]<- S[i,2,3]

S[i,2,4]<- control[i]*sqrt(S[i,2,2])*sqrt(S[i,4,4])/2

S[i,4,2]<- S[i,2,4]

S[i,4,3]<- control[i]*rho1*sqrt(S[i,4,4])*sqrt(S[i,3,3])

S[i,3,4]<- S[i,4,3] }

for (k in 1:(Ns-N2h)){

prec3A[k,1:4,1:4]<-inverse(S[k,,])

y[2*N2h+4*k-3:2*N2h+4*k]~dmnorm(mean[2*N2h+4*k-3:2*N2h+4*k],prec3A[k,,])}

*# Parameterization of the means*

for(i in 1:N2h) {

mean[2*i-1] <- -dR[T2[i]] + dR[T1[i]]

mean[2*i] <- -dD[T2[i]]+ dD[T1[i]]}

for(i in 1:(Ns-N2h)) {

mean[2*N2h+4*i-3] <- -dR[T2[N2h+i]] + dR[T1[N2h+i]]

mean[2*N2h+4*i-2] <- -dD[T2[N2h+i]] + dD[T1[N2h+i]]

mean[2*N2h+4*i-1] <- -dR[T3[N2h+i]]+ dR[T1[N2h+i]]

mean[2*N2h+4*i] <- -dD[T3[N2h+i]] + dD[T1[N2h+i]] }

*# Priors*

for(k in 1:(ref-1)) { dR[k] ~ dnorm(0,.01)}

for(k in (ref+1):NT) {dR[k] ~ dnorm(0,.01)}

for(k in 1:(ref-1)) { dD[k] ~ dnorm(0,.01)}

for(k in (ref+1):NT) {dD[k] ~ dnorm(0,.01)}

psiR.sq<-psi1*psi1

psi1~dunif(0,1)

psiD.sq<-psi2*psi2

psi2~dunif(0,1)

rho1~dunif(-1,0)

*#Estimated Effect Sizes*

dR[ref]<- 0

for (c in 1:(ref-1)) { Eff.refR[c]<- exp(dR[c] - dR[ref] )}

for (c in (ref+1):NT) { Eff.refR[c]<- exp(dR[c] - dR[ref] ) }

for (c in 1:(NT-1)) {

for (k in (c+1):NT) {

EffR[c,k] <- exp(dR[k] - dR[c])}}

dD[ref]<- 0

for (c in 1:(ref-1)) { Eff.refD[c]<- exp(dD[c] - dD[ref] )}

for (c in (ref+1):NT) { Eff.refD[c]<- exp(dD[c] - dD[ref] ) }

for (c in 1:(NT-1)) {

for (k in (c+1):NT) {

EffD[c,k] <- exp(dD[k] - dD[c]) }}

*# SUCRA rankings*

*# Ranking of treatments for response. This part of the code is adjusted for the acute mania*

*# dataset*

for (k in 1:13){ddR[k]<-dR[k]}

for(k in 1:13) {

orderR[k]<-13- rank(ddR[],k)

most.effectiveR[k]<-equals(orderR[k],1)

for(j in 1: 13) {

effectivenessR[k,j]<- equals(orderR[k],j)

cumeffectivenessR[k,j]<- sum(effectivenessR[k,1:j])}}

for(k in 1:13) {SUCRAR[k]<- sum(cumeffectivenessR[k,1:(13-1)]) /(13-1)}

*#Ranking of treatments for dropout*

for(k in 1:NT) {

orderD[k]<- rank(dD[],k)

most.effectiveD[k]<-equals(orderD[k],1)

for(j in 1: NT) {

effectivenessD[k,j]<- equals(orderD[k],j)

cumeffectivenessD[k,j]<- sum(effectivenessD[k,1:j])}}

for(k in 1:NT) {

SUCRAD[k]<- sum(cumeffectivenessD[k,1:( NT-1)]) /(NT-1)}}

The data needed as inputs for this program are y, varr, vard and T1, T2, T3, described in the end of the previous Section.

# RESULTS

In this Section we present the results for each model presented in the main paper. In Figure 2 the ORs for the response outcome of treatment vs placebo are presented, in Figure 3 for dropout. In Table 3 we present the rankings for all treatments based on their SUCRA values for each model.

*Table 3. Treatment ranking for all models based on the SUCRA values for response ($\boldsymbol{R}$) and dropout ($\boldsymbol{D}$).*

| **I** | | | | **II.a** | | | |
| --- | --- | --- | --- | --- | --- | --- | --- |
| **SUCRA – $\boldsymbol{R}$ (%)** | | **SUCRA – $\boldsymbol{D}$ (%)** | | **SUCRA – $\boldsymbol{R}$ (%)** | | **SUCRA – $\boldsymbol{D}$ (%)** | |
| Carbamazepine | 79.6 | Olanzapine | 91.7 | Haloperidol | 76.6 | Olanzapine | 91.2 |
| Haloperidol | 75.8 | Paliperidone | 86.3 | Olanzapine | 76.5 | Paliperidone | 87.5 |
| Olanzapine | 72.1 | Quetipaine | 80.3 | Carbamazepine | 75.2 | Quetipaine | 81.4 |
| Paliperidone | 69.0 | Divalproex | 68.8 | Paliperidone | 74.9 | Divalproex | 68.1 |
| Quetipaine | 60.4 | Aripiprazole | 65.6 | Aripiprazole | 62.4 | Aripiprazole | 65.9 |
| Aripiprazole | 60.1 | Carbamazepine | 57.6 | Quetipaine | 61.5 | Carbamazepine | 53.3 |
| Divalproex | 59.6 | Haloperidol | 52.9 | Asenapine | 54.4 | Haloperidol | 52.5 |
| Lithium | 47.1 | Ziprasidone | 45.1 | Divalproex | 54.0 | Ziprasidone | 46.5 |
| Asenapine | 45.2 | Asenapine | 39.7 | Lithium | 40.8 | Asenapine | 40.1 |
| Lamotrigine | 39.1 | Placebo | 34.1 | Ziprasidone | 27.9 | Placebo | 34.1 |
| Ziprasidone | 25.5 | Lithium | 32.7 | Lamotrigine | 26.5 | Lithium | 33.5 |
| Placebo | 9.7 | Lamotrigine | 23.5 | Topiramate | 9.9 | Lamotrigine | 24.3 |
| Topiramate | 6.9 | Gabapentin | 12.7 | Placebo | 9.4 | Gabapentin | 12.6 |
|  | | Topiramate | 9.1 |  | | Topiramate | 9.0 |
| **II.b** | | | | **II.c** | | | |
| **SUCRA – $\boldsymbol{R}$ (%)** | | **SUCRA – $\boldsymbol{D}$ (%)** | | **SUCRA – $\boldsymbol{R}$ (%)** | | **SUCRA – $\boldsymbol{D}$ (%)** | |
| Olanzapine | 77.9 | Olanzapine | 91.3 | Haloperidol | 78.5 | Olanzapine | 91.0 |
| Haloperidol | 77.0 | Paliperidone | 87.3 | Olanzapine | 78.4 | Paliperidone | 87.1 |
| Paliperidone | 76.4 | Quetipaine | 81.5 | Paliperidone | 77.1 | Quetipaine | 81.3 |
| Carbamazepine | 72.0 | Aripiprazole | 66.0 | Carbamazepine | 66.5 | Aripiprazole | 66.1 |
| Aripiprazole | 63.2 | Divalproex | 65.2 | Aripiprazole | 64.4 | Divalproex | 62.6 |
| Quetipaine | 61.8 | Carbamazepine | 57.1 | Quetipaine | 62.2 | Carbamazepine | 61.6 |
| Asenapine | 56.0 | Haloperidol | 51.7 | Divalproex | 56.6 | Haloperidol | 52.1 |
| Divalproex | 54.4 | Ziprasidone | 46.9 | Asenapine | 56.3 | Ziprasidone | 45.6 |
| Lithium | 42.2 | Asenapine | 39.9 | Lithium | 43.0 | Asenapine | 39.6 |
| Ziprasidone | 27.7 | Placebo | 34.1 | Ziprasidone | 28.3 | Placebo | 34.4 |
| Lamotrigine | 21.5 | Lithium | 33.7 | Lamotrigine | 17.8 | Lithium | 33.7 |
| Topiramate | 10.2 | Lamotrigine | 23.8 | Topiramate | 10.9 | Lamotrigine | 23.7 |
| Placebo | 9.7 | Gabapentin | 12.6 | Placebo | 10.0 | Gabapentin | 12.3 |
|  | | Topiramate | 9.0 |  | | Topiramate | 9.1 |
| **II.d** | | | | **III** | | | |
| **SUCRA – $\boldsymbol{R}$ (%)** | | **SUCRA – $\boldsymbol{D}$ (%)** | | **SUCRA – $\boldsymbol{R}$ (%)** | | **SUCRA – $\boldsymbol{D}$ (%)** | |
| Olanzapine | 76.5 | Olanzapine | 91.2 | Haloperidol | 78.1 | Olanzapine | 92.8 |
| Haloperidol | 75.4 | Paliperidone | 87.6 | Olanzapine | 74.1 | Paliperidone | 86.6 |
| Carbamazepine | 75.3 | Quetipaine | 81.7 | Paliperidone | 73.0 | Quetipaine | 80.6 |
| Paliperidone | 75.1 | Divalproex | 67.3 | Carbamazepine | 71.4 | Aripiprazole | 66.1 |
| Aripiprazole | 62.4 | Aripiprazole | 66.2 | Aripiprazole | 63.4 | Divalproex | 65.8 |
| Quetipaine | 61.8 | Carbamazepine | 53.0 | Quetipaine | 63.2 | Carbamazepine | 58.7 |
| Asenapine | 55.1 | Haloperidol | 52.7 | Asenapine | 56.0 | Haloperidol | 52.2 |
| Divalproex | 53.6 | Ziprasidone | 46.8 | Divalproex | 55.4 | Ziprasidone | 45.4 |
| Lithium | 41.5 | Asenapine | 39.4 | Lithium | 44.5 | Asenapine | 42.9 |
| Ziprasidone | 27.4 | Placebo | 34.3 | Ziprasidone | 29.1 | Lithium | 36.1 |
| Lamotrigine | 26.7 | Lithium | 33.9 | Lamotrigine | 21.6 | Placebo | 34.6 |
| Topiramate | 9.9 | Lamotrigine | 23.9 | Placebo | 10.1 | Lamotrigine | 15.1 |
| Placebo | 9.4 | Gabapentin | 12.8 | Topiramate | 10.1 | Gabapentin | 13.3 |
|  | | Topiramate | 9.2 |  | | Topiramate | 9.9 |

*Figure 2. Summary odds ratios for response, Treatment vs. Placebo for all scenarios presented in the paper.*


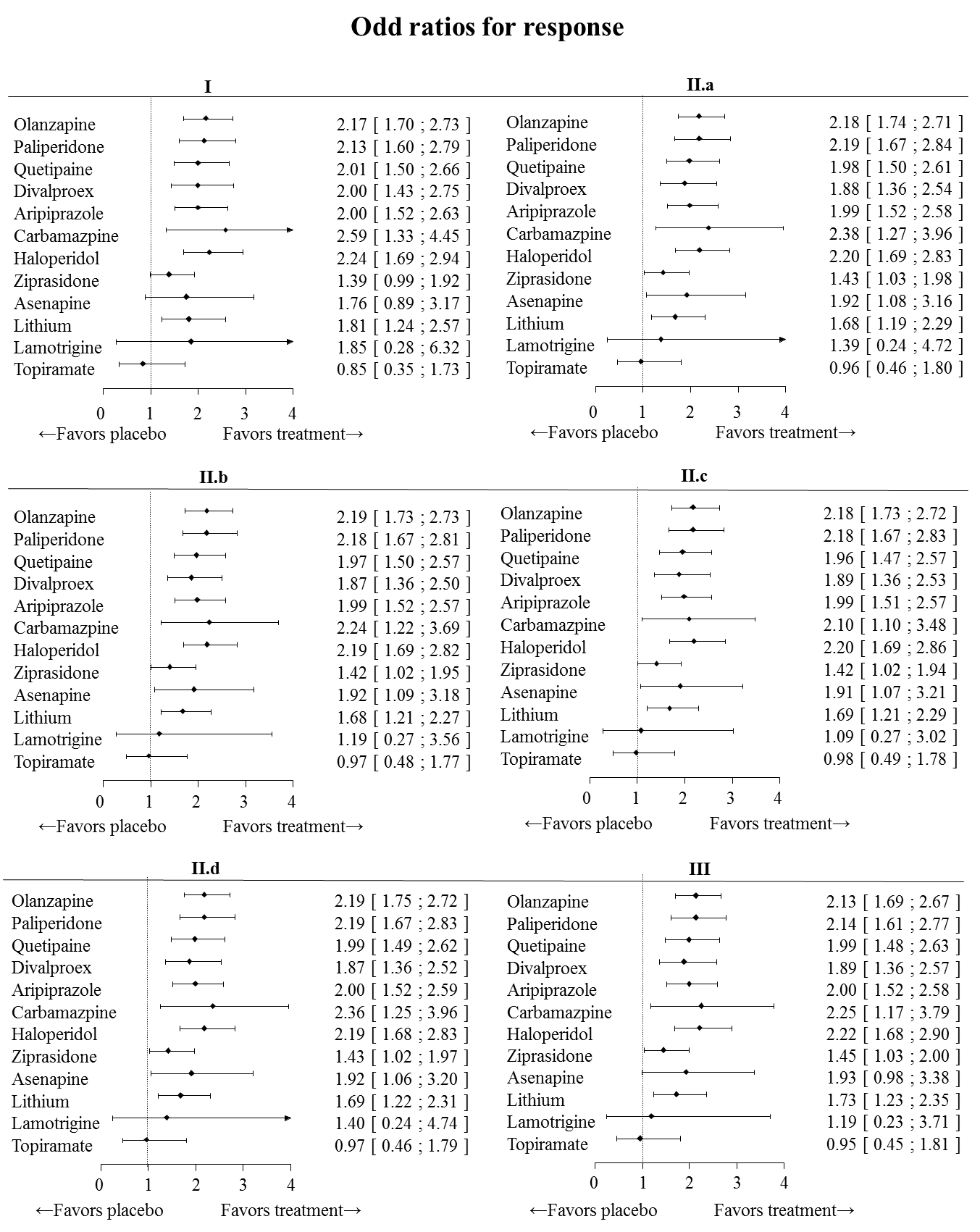


*Figure 3. Summary odds ratios for dropout, Treatment vs. Placebo for all scenarios presented in the paper.*


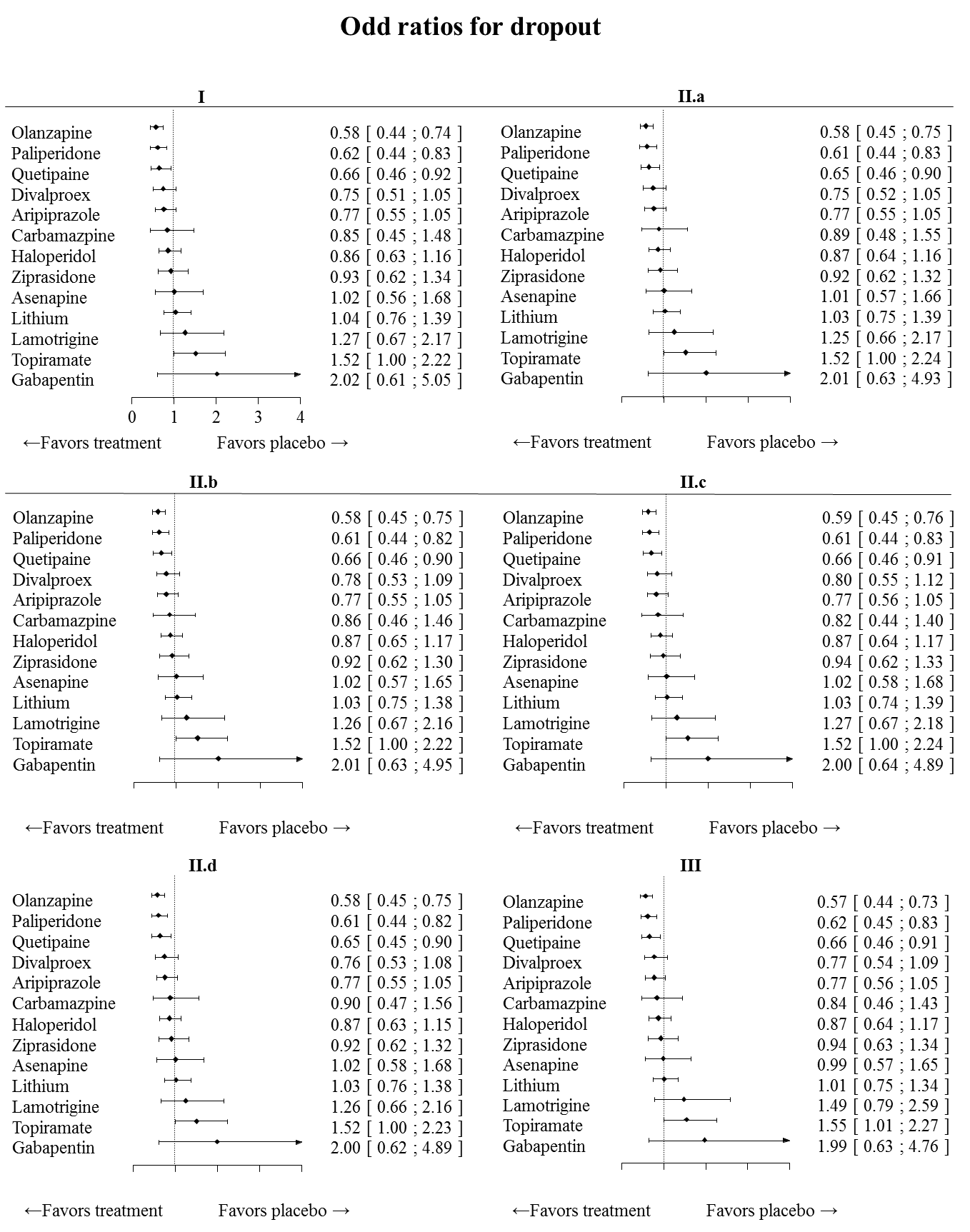


# EXTENDING THE MODELS

In this section we present ways to extend the two models proposed in this paper for the case of studies with more than three arms, reporting on more than two correlated outcomes of interest.

## *Generalizing the first model*

We start from the case of pairwise meta-analysis, when only two treatments are compared for three outcomes. Suppose there are studies reporting on a single comparison $A$ versus $B$, for three correlated outcomes $R$, $D$ and $V$. The random errors for every study are assumed to follow a multivariate normal distribution ${(\delta}_{R} \delta_{D} \delta_{V})' \sim N(0,\boldsymbol{\Delta}_{\left( \boldsymbol{3\times3} \right)})$, with variance-covariance matrix:

$$\text{Δ}_{\text{(3×3)}}=\left(\begin{matrix} \tau_{R}^{2} & . & . \\ \rho_{RD}^{\tau}\tau_{R}\tau_{D} & \tau_{D}^{2} & . \\ \rho_{RV}^{\tau}\tau_{R}\tau_{D} & \rho_{DV}^{\tau}\tau_{R}\tau_{D} & \tau_{V}^{2} \end{matrix}\right)$$

Note that there are in principle three heterogeneities and three different between-study correlation coefficients that need to be estimated.

The random errors of study $i$ are also assumed to follow a multivariate normal distribution ${(\varepsilon}_{i,R} \varepsilon_{i,D} \varepsilon_{i,V})' \sim N(0,\boldsymbol{\Sigma}_{\boldsymbol{i}})$. The within-study variance-covariance matrix is:

$$\text{Σ}_{\text{i}}=\left(\begin{matrix} \sigma_{i,R}^{2} & . & . \\ \rho_{i,RD}\sigma_{i,R}\sigma_{i,D} & \sigma_{i,D}^{2} & . \\ \rho_{i,RV}\sigma_{i,R}\sigma_{i,D} & \rho_{i,DV}\sigma_{i,R}\sigma_{i,D} & \sigma_{i,V}^{2} \end{matrix}\right)$$

Thus, there are also three different within-study correlation coefficients to estimate.

We now extend the method for the case of multi-arm studies reporting on three correlated outcomes for a multiplicity of treatments. If we focus on a three-arm study, the heterogeneity variance-covariance matrix will be a $(6\times6)$ generalization of the matrix of Equation :

$$\text{Δ}_{\text{(6×6)}}=\left(\begin{matrix} \tau_{R}^{2} & . & . & . & . & . \\ \rho_{RD}^{\tau}\tau_{R}\tau_{D} & \tau_{D}^{2} & . & . & . & . \\ \rho_{RV}^{\tau}\tau_{R}\tau_{V} & \rho_{DV}^{\tau}\tau_{D}\tau_{V} & \tau_{V}^{2} & . & . & . \\ \tau_{R}^{2}/2 & \rho_{RD}^{\tau}\tau_{R}\tau_{D} & \rho_{RV}^{\tau}\tau_{R}\tau_{V} & \tau_{R}^{2} & . & . \\ \rho_{RD}^{\tau}\tau_{R}\tau_{D} & \tau_{D}^{2}/2 & \rho_{DV}^{\tau}\tau_{D}\tau_{V} & \rho_{RD}^{\tau}\tau_{R}\tau_{D} & \tau_{D}^{2} & . \\ \rho_{RV}^{\tau}\tau_{R}\tau_{V} & \rho_{DV}^{\tau}\tau_{D}\tau_{V} & \tau_{V}^{2}/2 & \rho_{RV}^{\tau}\tau_{R}\tau_{V} & \rho_{DV}^{\tau}\tau_{R}\tau_{D} & \tau_{V}^{2} \end{matrix}\right)$$

The within-study variance-covariance matrix for this study, after making the same simplifying assumptions as in Section 0 of this Appendix, can be estimated as follows:

$$\text{S}_{\text{i}}=\left(\begin{matrix} s_{i,AB,R}^{2} & . & . & . & . & . \\ \rho_{i,RD} & s_{i,AB,D}^{2} & . & . & . & . \\ \rho_{i,RV} & \rho_{i,DV} & s_{i,AB,V}^{2} & . & . & . \\ \kappa_{i,AB_{R}AC_{R}} & 0.5\rho_{i,RD} & 0.5\rho_{i,RV} & s_{i,AC,R}^{2} & . & . \\ 0.5\rho_{i,RD} & \kappa_{i,AB_{D}AC_{D}} & 0.5\rho_{i,VD} & \rho_{i,RD} & s_{i,AC,D}^{2} & . \\ 0.5\rho_{i,RV} & 0.5\rho_{i,VD} & \kappa_{i,AB_{V}AC_{V}} & \rho_{i,RV} & \rho_{i,DV} & s_{i,AC,V}^{2} \end{matrix}\right)$$

In the above we have dropped the standard errors that multiply the correlation coefficients for simplicity. We now have three different within-study correlation coefficients to estimate for every three-arm study. As before, we can model these coefficients to be common across studies or among group of studies.

Extending for more arms or more outcomes is straightforward. For example, a four-arm study in the case of three outcomes of interest will require a $9\times9$ generalization of the above matrices*.*

## *Generalizing the second model*

In this subsection we will show how to extend the second model presented in Section 3.2.2 of the main paper for the case of more than two correlated outcomes, or in the presence of studies than more than three arms. Let us start by assuming a network of studies reporting on three outcomes $R$, $D$ and $V$. For a two-arm study the variance-covariance matrix can be estimated as:

$$\text{Ω}_{i}=\left(\begin{matrix} z_{i,AB,R} & . & . \\ \rho_{RD}^{h}\sqrt{z_{i,AB,R}z_{i,AB,D}} & z_{i,AB,D} & . \\ \rho_{RV}^{h}\sqrt{z_{i,AB,R}z_{i,AB,V}} & \rho_{DV}^{h}\sqrt{z_{i,AB,D}z_{i,AB,V}} & z_{i,AB,V} \end{matrix}\right)$$

Note that we now need three different hybrid correlation coefficients to be estimated from the model. For a three-arm study comparing treatments $A$, $B$ and $C$ for three outcomes $R$, $D$ and $V$ a $\left( 6\times6 \right)$ variance-covariance matrix is needed instead:

$$\text{Ω}_{i}=\left(\begin{matrix} z_{i,AB,R} & . & . & . & . & . \\ \rho_{RD}^{h} & z_{i,AB,D} & . & . & . & . \\ \rho_{RV}^{h} & \rho_{DV}^{h} & z_{i,AB,V} & . & . & . \\ 1/2 & \rho_{RD}^{h}/2 & \rho_{RV}^{h}/2 & z_{i,AC,R} & . & . \\ \rho_{RD}^{h}/2 & 1/2 & \rho_{DV}^{h}/2 & \rho_{RD}^{h} & z_{i,AC,D} & . \\ \rho_{RV}^{h}/2 & \rho_{DV}^{h}/2 & 1/2 & \rho_{RV}^{h} & \rho_{DV}^{h} & z_{i,AC,V} \end{matrix}\right)$$

In the above matrix we have dropped the $z$ parameters in the elements off the diagonal, for simplicity. We can generalize for the case of studies with more arms, and for multiple outcomes by following the same pattern. Note that care should be taken so that all the variance-covariance matrices presented in this section remain definite-positive. In Section 3 of the main paper and Section 4 of this Appendix we discussed ways to ensure the positive-definiteness.

**References**

Chaimani, A., J.P.T. Higgins, D. Mavridis, P. Spyridonos, and G. Salanti
 2013 Graphical Tools for Network Meta-Analysis in STATA. PLoS.One. 8(10): e76654.

Franchini, A.J., S. Dias, A.E. Ades, J.P. Jansen, and N.J. Welton
 2012 Accounting for Correlation in Network Meta-Analysis with Multi-Arm Trials. Res.Synth.Meth. 3(2): 142–160.

Gleser, LJ, and Larry V. Olkin
 2009 Stochastically Dependent Effect Sizes. In The Handbook of Research Synthesis and Meta-Analysis, (Cooper H, Hedges LV (eds). Russell Sage Foundation.

Riley, Richard D., John R. Thompson, and Keith R. Abrams
 2008 An Alternative Model for Bivariate Random-Effects Meta-Analysis When the within-Study Correlations Are Unknown. Biostatistics 9(1): 172–186.
